# Systematic review of the use of ultrasound for venous assessment and venous thrombosis screening in spaceflight

**DOI:** 10.1038/s41526-024-00356-w

**Published:** 2024-02-05

**Authors:** Antoine Elias, Tobias Weber, David A. Green, Katie M. Harris, Jonathan M. Laws, Danielle K. Greaves, David S. Kim, Lucia Mazzolai-Duchosal, Lara Roberts, Lonnie G. Petersen, Ulrich Limper, Andrej Bergauer, Michael Elias, Andrew Winnard, Nandu Goswami

**Affiliations:** 1 Cardiology and Vascular Medicine, Sainte Musse Hospital, Toulon Hospital Centre, Toulon, France; 2 Clinical Research and Innovation, Sainte Musse Hospital, Toulon Hospital Centre, Toulon, France; 3 Investigation Network On Venous Thrombo-Embolism | French Clinical Research Infrastructure Network (INNOVTE | F-CRIN), Toulon, France; 4 Space Medicine Team (HRE-OM), European Astronaut Center (EAC), European Space Agency (ESA), Cologne, Germany; 5 KBR, Cologne, Germany; 6 Centre of Human and Applied Physiological Sciences, King’s College London, London, United Kingdom; 7 Faculty of Medicine, Memorial University of Newfoundland, St. John’s, NL Canada; 8 University of Northumbria at Newcastle, Newcaslte-upon-Tyne, United Kingdom; 9 Space Biomedicine Systematic Review Methods Group, Wylam, United Kingdom; 10 Faculty of Health, University of Waterloo, Waterloo, ON Canada; 11 Department of Emergency Medicine, Faculty of Medicine, University of British Columbia, Vancouver, BC Canada; 12 Department of Angiology, Lausanne University, Lausanne, Switzerland; 13 King’s Thrombosis Centre, Department of Haematological Medicine, King’s College Hospital NHS Foundation Trust, London, United Kingdom; 14 Institute of Pharmaceutical Sciences, King’s College London, London, United Kingdom; 15 Department of Aeronautics and Astronautics, Massachusetts Institute of Technology, Cambridge, MA USA; 16 German Aerospace Center (DLR), Institute of Aerospace Medicine, Cologne, Germany; 17 University of Witten / Herdecke, Department of Anaesthesiology and Critical Care Medicine, Merheim Medical Center, Hospitals of Cologne, Cologne, Germany; 18 Department of Surgery, LKH Südsteiermark, Wagna, Austria; 19 Gravitational Physiology and Medicine Research Unit, Division of Physiology, Otto Loewi Research Center, Medical University of Graz, Graz, Austria; 20 Critical Care Medicine, St. Vincent’s Medical Center, Hartford Healthcare, Bridgeport, CT USA; 21 The Frank H. Netter MD School of Medicine, North Haven, CT USA; 22 Division of Physiology, Otto Loewi Research Center of Vascular Biology, Immunity and Inflammation, Medical University of Graz, Graz, Austria; 23 Mohammed Bin Rashid University of Medicine and Applied Health Sciences, Dubai, United Arab Emirates

**Keywords:** Diagnostic markers, Thrombosis, Ultrasonography

## Abstract

The validity of venous ultrasound (V-US) for the diagnosis of deep vein thrombosis (DVT) during spaceflight is unknown and difficult to establish in diagnostic accuracy and diagnostic management studies in this context. We performed a systematic review of the use of V-US in the upper-body venous system in spaceflight to identify microgravity-related changes and the effect of venous interventions to reverse them, and to assess appropriateness of spaceflight V-US with terrestrial standards. An appropriateness tool was developed following expert panel discussions and review of terrestrial diagnostic studies, including criteria relevant to crew experience, in-flight equipment, assessment sites, ultrasound modalities, and DVT diagnosis. Microgravity-related findings reported as an increase in internal jugular vein (IJV) cross-sectional area and pressure were associated with reduced, stagnant, and retrograde flow. Changes were on average responsive to venous interventions using lower body negative pressure, Bracelets, Valsalva and Mueller manoeuvres, and contralateral IJV compression. In comparison with terrestrial standards, spaceflight V-US did not meet all appropriateness criteria. In DVT studies (*n* = 3), a single thrombosis was reported and only ultrasound modality criterion met the standards. In the other studies (*n* = 15), all the criteria were appropriate except crew experience criterion, which was appropriate in only four studies. Future practice and research should account for microgravity-related changes, evaluate individual effect of venous interventions, and adopt Earth-based V-US standards.

## Introduction

There is a growing interest in studying deep venous thrombosis (DVT) in space since the first publications reporting on the coincidental detection of a thrombosis in the internal jugular vein (IJV) on board the International Space Station (ISS)^1,2^. The IJV thrombosis was reported as a possible consequence of venous congestion and blood flow anomalies in relation to fluid cephalad shifts, in addition to other venous thromboembolism (VTE) risk factors^3^.

Whilst DVT appears to be rare in space, it is a relevant issue because of its potential seriousness given it can affect cerebral drainage and/or cause pulmonary embolism, and because of the difficulty of remote diagnostic and therapeutic management. Such challenges are only going to increase as missions go beyond Low Earth Orbit (LEO) as the restrictions on mass, volume, re-supply and communications become more severe^4^. In addition, whilst at present astronauts are highly selected, commercial spaceflight will involve consideration of less stringent medical requirements^5^.

One of the main obstacles to the management of VTE in space is its diagnosis. Whilst ultrasound (US) is the preferred imaging modality both on the ISS and terrestrially, several questions remain unanswered regarding its use for the definitive diagnosis of DVT in space. The first is its validity and the risk of diagnostic error, particularly false positives, given the similarity of the thrombosis-related aspects with those induced by microgravity (i.e., venous congestion and flow stasis)^6^. The second is the actual extent and therefore the possible deep involvement e.g., at the level of the innominate venous trunk and the intra-thoracic veins. The third issue is the lack of studies on diagnostic accuracy and diagnostic management in patients with clinically suspected DVT in space. Therefore, the optimal venous ultrasound (V-US) criteria to rule-out and conversely rule-in the diagnosis of DVT in space remain to be defined, as is the case with clinical presentation including VTE risk factors^7^, and D-dimer and endothelial biomarkers^8^.

Given the lack of diagnostic studies, we sought to objectively and comprehensively explore how V-US was used and interpreted in spaceflight and thus conducted a systematic review of the studies reporting V-US in space to identify approaches in microgravity and compare them to terrestrial clinical best-practice V-US.

The objective of this review was to gather data to help inform practice, research, and guide recommendations for technical implementation of V-US in space to achieve an accurate diagnosis of DVT with better discrimination between DVT and microgravity-related congestion phenomenon in the upper body venous system.

This raised the following questions:

- What are the changes related to microgravity, particularly those that may confound the diagnosis of DVT, to account for when performing V-US in spaceflight?
- How to reverse microgravity-related changes and restore normal terrestrial conditions for optimal venous assessment in spaceflight?
- How was V-US performed in spaceflight and to what extent was it appropriate for DVT detection in comparison with terrestrial V-US?
- What are the implications for practice and research?

## Methods

The methodological approach and reporting of the results followed the Preferred Reporting Items for Systematic Reviews and Meta-Analyses (PRISMA) statement guidelines^9,10^ and the Space Biomedicine Systematic Review Methods (https://sites.google.com/view/sr-methods/guides)^11^. The protocol was registered in PROSPERO database (Registration number [CRD42023410886]).

### Selection criteria

All four following criteria had to be met to include a publication for analysis in the systematic review: (1) the study had to have been carried-out in human participants, (2) in the setting of short or long-term spaceflights or acute exposure to microgravity, (3) using an US method including B-mode US, Doppler US, duplex US or Colour-Doppler US modality and (4) reporting any US assessment of the neck/ upper limb or lower limb venous systems (Table 1). Thus, studies on animals, human studies on ground-based spaceflight analogues, reviews, and duplicates (i.e., same record found in different databases) were excluded. The type of study whether observational (descriptive or comparative) or interventional was not a criterion for considering studies for this review. If a study was published more than once (multiple records), the earliest published article or the article combining cumulative results from different studies were included.

**Table 1:** Research question in PICOS Format.

PICOS items	Search type
Participants	Human participants during spaceflights
Interventions	Spaceflight venous ultrasound (short-term or long-term spaceflights, or acute exposure to microgravity)
Comparison	Terrestrial venous ultrasound
Outcomes	Venous ultrasound morphological and physiological changes during spaceflights
	Effect of inflight countermeasures/ interventions on venous ultrasound changes
	Gaps and technical issues when performing spaceflight venous ultrasound (i.e., appropriateness of spaceflight venous ultrasound)
Study design	Observational (descriptive or comparative) and interventional studies

### Search methods for identification of studies

Following databases were systematically searched for relevant studies: PubMed (National Institutes of Health, National Library of Medicine), OVID versions of MEDLINE, Embase, Cochrane Library of Databases and EBM Reviews, since inception to July 24^th^, 2022. Google Scholar and databases maintained on NASA and ESA websites (https://www.nasa.gov/centres/hq/library/find/databases), were checked for pending or recently published full articles or conference presentations. Further searches included the reference lists of relevant articles and reviews, and citation index of journal websites and databases. The search also covered various sources of ‘grey literature’ (mainly conference abstracts and protocols) to identify published, unpublished, and ongoing studies. We also contacted study authors for unavailable (missing), incomplete or unclear study results.

There were no date or language restrictions on the searches. Languages other than English and French were translated from their original language to English by professional scientific translators.

The following Medical Subject Headings (MESH) and text words search terms were used:

1. “microgravity” OR “spaceflight” OR “hypogravity” OR “reduced gravity” OR “zero gravity” OR “weightlessness” OR “micro-G” OR “low gravity” OR “zero-G” OR “astronaut*” OR “cosmonaut”
2. “ultrasound*” OR “B mode” OR “echography” OR “sonograph*” OR “doppler”
3. “vein*” OR “venous*” OR “DVT” OR “VTE”.

These were then combined as shown in Table 2.

**Table 2:** Search method.

Category	Query	Search number	Search field
Microgravity	microgravity	1	All fields
	spaceflight	2	All fields
	hypogravity	3	All fields
	reduced gravity	4	All fields
	zero gravity	5	All fields
	weightlessness	6	All fields
	micro-G	7	All fields
	low gravity	8	All fields
	zero-G	9	All fields
	astronaut*	10	All fields
	cosmonaut*	11	All fields
	#1 OR #2 OR #3 OR #4 OR #5 OR #6 OR #7 OR #8 OR #9 OR #10 OR #11	12	
Ultrasound	ultrasound	13	All fields
	B?mode	14	All fields
	echography	15	All fields
	sonograph*	16	All fields
	doppler	17	All fields
	#13 OR #14 OR #15 OR #16 OR #17	18	
Venous assessment	vein	19	All fields
	venous	20	All fields
	vte	21	All fields
	dvt	22	All fields
	#19 OR #20 OR #21 OR #22	23	
All combined	#12 AND #18 AND #23	24	

### Selection of studies

After having removed duplicates, potentially eligible studies were identified by examining titles and abstracts, with full articles acquired when appropriate. Study assessment was performed by two independent and blinded reviewers to avoid selection bias. Disagreements were all resolved through discussion between the assessors.

### Data extraction

First, data was extracted from selected studies regarding study characteristics: author, year of publication, participating centres, time-period when study was conducted, study duration, objective, design, population, outcome measures, analysis issues and summary of findings.

Second, data was extracted about V-US changes in-flight for the neck/ upper limb venous system, to establish a baseline for the diagnostic exclusion of DVT and prevent DVT overdiagnosis and overtreatment, with a special focus on DVT screening studies. Data was also extracted about the type and the effect of venous interventions on such changes, including lower body negative pressure (LBNP) at different negative pressure levels, thigh-cuffs (Bracelets or “Braslets”), respiratory manoeuvres (Valsalva and Mueller manoeuvres), and contralateral IJV compression.

Finally, to assess appropriateness of spaceflight V-US with terrestrial V-US standards, we performed a review regarding methods and performances of V-US in diagnostic accuracy and diagnostic management studies conducted on Earth in patients with clinically suspected upper extremity deep vein thrombosis (see methods in Supplementary Materials). A bias assessment tool based on terrestrial V-US standards was developed following discussions with a group of experts in space medicine, vascular medicine, venous thrombosis, and vascular ultrasound. The tool included appropriateness criteria for an optimal V-US relevant to:

1. crew experience: individuals who performed and interpreted the V-US (expert physician or sonographer astronaut or non-expert, use of remote control or remote guidance, personnel training, image captures, annotation procedures and V-US interpretation),
2. in-flight equipment: the types of equipment used for V-US assessment (US device/ scanner, probe emission frequency for peripheral and central vein studies, US modalities),
3. assessment sites: the sites examined for V-US assessment (right side, left side or both, vein segments and US views, for neck/ upper limb venous systems).
4. US methods: the US diagnostic methods used (B-mode, Colour-Doppler, Duplex US, …) for the assessment of the neck/ upper limb system, and
5. DVT diagnosis: the diagnostic criteria used for DVT screening if any.

For each type of data collected on V-US characteristics in spaceflight studies, we attempted to capture when in-flight V-US was comparable to terrestrial state-of-the-art clinical practice and when it was not.

Data (Supplementary Table 1) was collected using standardised forms on Excel sheet.

### Data analysis and synthesis

Our objective was to perform a descriptive analysis of study characteristics and V-US assessment characteristics, and to provide a summary of findings regarding in-flight changes, effect of venous interventions on these changes, and V-US assessment characteristics with a special focus on studies reporting on DVT diagnosis.

We did not report a quantitative synthesis of the changes and the effects of interventions as this was not the primary objective and because of an important heterogeneity between studies.

### Reporting summary

Further information on research design is available in the Nature Research Reporting Summary linked to this article.

## Results

### Search results

**Fig. 1:**
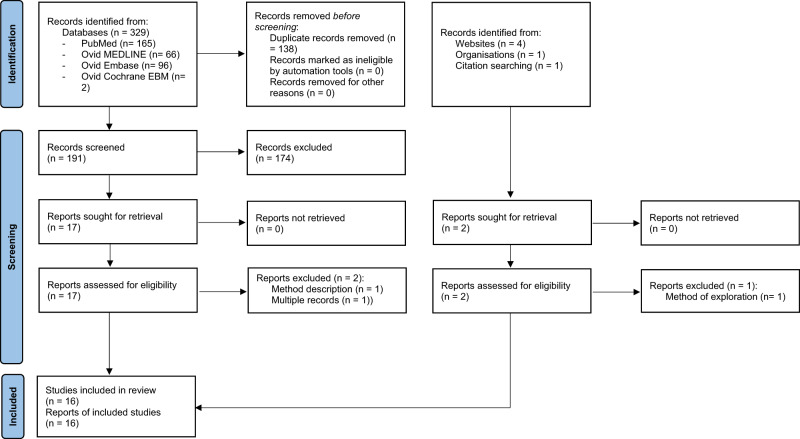
Search strategy based on PRISMA 2020 flow diagram^10^. Records were identified from databases and other sources, then screened and assessed for eligibility.

Figure 1 shows the flow of studies through the review following PRISMA 2020 updated guideline^10^.

Based on our search criterion through electronic databases (Supplementary Tables 2–5): 165 records were obtained in PubMed, 66 in MEDLINE, 96 in Embase and 2 in Cochrane and EBM reviews yielding a total of 329 records Many records were duplicates (*n* = 138), were not eligible (*n* = 174) with all three eligibility criteria being unmet (*n* = 94), two of them unmet (*n* = 25), or only one unmet (*n* = 49) and other reasons (*n* = 6) such as records published in forms of abstract (*n* = 1), review (*n* = 2), reply/ comments (*n* = 1) and multiple records (*n* = 2). Thus, finally, 17 full text records were sought for retrieval and assessed for eligibility of which two were excluded (one a method description about remote echography^12^ and the other a review paper^13^), but 15 were retained^1,2,14–26^.

Six other studies were identified via the other sources provided in “Search methods” section of which two were retrieved and assessed for eligibility. One was excluded because it did not use the ultrasound as assessment method^27^ but the other one was retained^28^.

Therefore, 16 studies in total were included in present analysis.

### Characteristics of included studies

Included studies were published between 1994 and 2022. Two reported on acute exposure to microgravity and hypogravity during parabolic flight campaigns^23,24^, one on short-term^28^ and ten on long-term (≥6 months) chronic exposure^2,14–16,18,19,21,22,25,26^; In three other studies the class of exposure duration was not reported^1,17,20^. Only three studies involved DVT detection^1,2,25^ of which one reported exclusively on DVT^1^ (Table 3).

**Table 3:** Characteristics of included studies.

Study author Study acronym	Participating centres Study time-period (TP) Study duration (D)	Study objective	Study design	Study population	Study outcomes/ measures Analysis issues	Summary of venous ultrasound findings
Arbeille et al.^28^ “Antarès” Spaceflight	On board MIR station Faculté de Médecine – Tours- France Institute of Biomedical Problems – Moscow-Russia TP: NR D: 89 days (pre-flight 60 days, in-flight 14 days and post-flight 15 days)	At rest: cardiovascular adaptation to 0 g at the level of the heart, the deep and superficial vessels. During LBNP (−25, −35, −45mmHg of 10 min): orthostatic tolerance by monitoring the cerebral and femoral response	Interventional Prospective study Repeated measurements - pre-flight (Days −60, −30, −10), in-flight (Days +5, +9, +12, and with the LBNP on day +11) - post-flight (at landing day +1, +3, +7, +15)	1 astronaut	IJV and femoral vein CSA, IJV distensibility, IJV and hepatic vein indices Blood pressure and CVP Calf volume changes measured by plethysmography. Analysis limited to a single astronaut	IJV CSA increased from D1 in-flight, and femoral vein after D9. LBNP: Disadapted response for the femoral vascular resistance
Arbeille et al.^14^	Unité de médecine et de Physiologie Spatiales Tours - France Institute of Biomedical Problems, Moscow-Russia TP: NR D: until 6 months	To identify the major cardiovascular changes to real or simulated 0 g with a minimum of countermeasures Summary of multiple studies performed by the author.	Interventional Prospective studies Repeated measurements (Spaceflights: 6, 14, 21, 25 days and 6 months. HDT: 10 h, 4, 5, 7, 30 and 42 days)	NR Review and update regarding multiple studies by the author	IJV and femoral vein CSA Wilcoxon matched pair test Sample size not provided	IJV CSA significantly enlarged during the flight and HDT (+40%). Femoral vein significantly enlarged during spaceflight after 1 week (+15% to +35%), significantly decreased after 4–5 days of HDT (−20% to −35%)
Arbeille et al.^15^ The Vessel Imaging study	ISS (NASA, ESA, CNES) TP: 2009–2013 D: 6 months	To investigate during a 6-month spaceflight the changes in the major central and peripheral vein properties, to quantify venous blood flow redistribution during the early and late phases of exposure to microgravity	Interventional Prospective cohort Repeated measurements -pre-flight (supine, seated) -in-flight (Day 15, 115, 135 and 15 days before return to earth) -post-flight (4 days after return)	10 astronauts Age: 47 ± 5 Gender: M/F 7/3 Mass: 69 ± 12 kg Height: 1.72 ± 8 m	CSA of the IJV, portal, femoral, gastrocnemius and tibial veins Volume of the IJV and the portal vein Ratio of IJV to portal vein volumes 2/10 excluded from inflight analysis for missing data	Increased IJV and portal vein volumes, increased femoral vein area, and reduced calf vein area. Significant venous blood pooling that persists throughout the duration of the spaceflight
Arbeille et al.^16^	ISS (NASA, UCSD) TP: NR D: 210 ± 76 days	To investigate microgravity-associated increase in IJV volume, portal vein CSA, and intracranial venous blood velocity and determine if LBNP would return variables toward pre-flight levels	Interventional Prospective cohort Repeated measurements with and without LBNP: -pre-flight (supine, seated, 15° HDT) -in-flight (Day 45, 150) -post-flight (40 and 180 days after return to earth)	14 astronauts Age: 47 ± 6 Gender: M/F 11/3 BMI: 26.4 ± 3	IJV volume Portal vein area Middle cerebral vein velocity LBNP −25 mmHg Some measurements not available at various time points (due to poor ultrasound image quality, inability to find the middle cerebral vein, inadequate images of the portal vein).	Increased IJV volume and portal vein area Increased middle cerebral vein velocity. Sustained cephalad fluid shift (head and splanchnic regions) and impact on cerebral venous circulation. LBNP −25 mmHg restored variables at least back to pre-flight supine levels.
Aunon et al.^1^	ISS TP: NR D: NR	Case report	Descriptive	1 patient	1/ Reporting a case of a left IJV thrombosis in spaceflight detected during a vascular research study in an asymptomatic patient	1/ Asymptomatic patient while the thrombus occlusive
					2/ US guided in real time and interpreted by two radiologists	2/ US performed by astronaut
					3/ Image of subacute (?) venous thrombosis reported	3/ Image compatible with a stagnant flow. No obvious direct image of the thrombus although a L12 MHz probe was used at 1 to 2 cm distance from skin.
					4/ US surveillance at 7-to-21-day intervalsshowed progressive organisation and volume reduction of the thrombus.	4/ Thrombus consistency pattern may depend on US compression test. The estimation of volume reduction may depend on probe positioning. No diameter or volume measurement was performed.
					5/ No spontaneous flow. Flow through the affected IJV segment was first noted on treatment day 47, but only on augmentation by Müller manoeuvre.	5/Flow on augmentation by Müller manoeuvre was detected through the affected IJV segment means this was a stagnant flow
					6/ The absence of spontaneous flow persisted after 90 days of anticoagulation but on landing (4 days after anticoagulation was stopped), a point-of-care US examination revealed spontaneous flow.	6/ How to explain the recovery of a spontaneous flow on landing (whereas it was absent during more than 90 days in space) other than by the abolition of stasis-related microgravity on landing.
David et al.^17^	NASA Gagarin Cosmonaut Training Centre TP : NR D : NR	To assess the effects of the long-used Russian (known as the Russian pre-launch) tilt-table training protocol on IJV CSA in microgravity	A case study	A single healthy male volunteer astronaut	Multiple measurements of the CSA at −15°, −30°, and +50°, along with 0° (pre-tilt) and 0° (post-tilt). The average CSA was computed for each angle and compared to the in-flight average using Student’s t-test.	Right IJV-CSA were significantly different between in-flight values and several angles of the Russian tilt-table protocol (higher with negative angles), except for the 0° measurement (post-tilt 0°).
Fomina et al.^18^	Flights of Russian and Russian-French crews on board Mir orbital station TP: 1994–1999 D: (438-day astronaut-doctor mission, 15–17, 22–23, 25–27 of the main expedition)	To analyse changes in human venous haemodynamic during prolonged exposure (6 months) to weightlessness	Interventional Prospective cohort Repeated measurements	7 astronauts for the US exam (six men) 33 to 52 years old	B-mode and doppler US - IJV CSA measurements before, during and after SF at rest and during a dosed Valsalva test (exhalation into a special mouthpiece connected to a manometer until a pressure of 50 mmHg was reached), - Femoral CSA measurements taken only at rest before, during and after SF. - Large abdominal veins (hepatic, portal and spleen veins) Occlusion plethysmography Analysis issues comparison of means (only 7 astronautrs for the US exam)	IJV CSA: significant increase by 28 ± 4.3% throughout the long-term SF, but no increasing trend in changes from month 1 to month 6. Wall distensibility did not increase (“increased IJV area most likely solely due to venous stasis”) Femoral area: significant increase by 31.3 ± 5.0% in the 1st month of SF; significant difference in femoral area between months 1 and 5–6 of SF (decrease in venous blood flow rate i.e. venous return) Abdominal veins (hepatic, portal and spleen veins) enlarged.
Fomina et al.^19^	Mir orbital space station (OSS) TP: NR D: 126 to 183 days (a physician cosmonaut on board for a 438-day mission)	To assess the effect of long-term use of prophylactic device (PD) Braslet on haemodynamic changes in a spaceflight lasting between 126 and 438 days	Interventional Prospective cohort Repeat measurements on the same day, after 5 h of Braslet use. Braslet during the flight in the daytime; Braslet-free period 10–12 h. Three investigations over 6 months in 6 men (a 38-year-old woman actively used PD Braslet only during the initial period of adaptation to weightlessness, and then only on the study day of the ECHO-Braslet experiment programme throughout the flight)	7 cosmonauts (six men) aged 33 to 52 years old	Changes in haemodynamic were assessed as a percentage of the pre-mission background and relative to the control without PD Braslet on the day of the study. Mean values were calculated for the group of cosmonauts who regularly used PD Braslet (six), and the mean error of the mean values (M ± t). “Difference in the mean in small samples, odds ratio (p), Student’s Distribution (t)”	Braslet-free: Reduced SV −17%, CO −22%, renal artery resistance without significant changes in blood pressure and heart rate, cephalic congestion (blood rush), IJV and femoral vein dilation, tendency for average cerebral blood flow rate to decrease slightly Braslet: SV and CO: No change at months 1 and 3, higher at month 5. Reduction of cephalic congestion and IJV CSA, no noticeable effect on cerebral blood flow, femoral vein CSA higher
Hamilton et al.^20^	ISS (NASA Telescience Centre in Houston, TX, and the Mission Control Centre in Korolyov, Moscow region, Russia) TP: NR D: NR	To examine the responses to modified Valsalva and Mueller manoeuvres measured by cardiac and vascular ultrasound (ECHO) in a baseline steady state and under the influence of thigh occlusion cuffs as a countermeasure device (Braslet cuffs)	Interventional Prospective cohort Repeated measurements during modified Valsalva and Mueller manoeuvres with and without thigh occlusion cuffs	9 astronauts Age: NR Gender: NR	Effect of acute application of Braslet on cardiac parameters, and IJV and Femoral vein CSA Comparison of Valsalva, Mueller, and baseline manoeuvres with and without Braslet High quality study and reporting	Braslet reduces the effective circulating volume by sequestering fluid in the lower extremities (increased femoral vein area) Braslet reduces preload indexes measured by echocardiography. Braslet reduces IJV distention, increases sensitivity of IJV area to thoracic manoeuvres.
Herault et al.^21^	MIR station (French and Russian): three 6-month spaceflights TP: NR D: 3 long-term 6-month spaceflights	To study the effects of thigh cuffs (braslets) on cardiovascular adaptation (cardiac, arterial, and venous changes) and deconditioning in 0 g.	Interventional Prospective cohort At rest: Repeated measurements (pre-flight D-30, inflight 1 month/3 months/5 months, postflight D1 and D7): before thigh cuffs, after 5 h with the thigh cuffs. LBNP −45 mmHg (without the cuffs) 1 day after each resting measurement session	6 astronauts	Cardiac parameters (LVEDV, SV, CO, ejection fraction) Arterial resistance: middle cerebral artery, carotid artery and renal artery resistance, femoral artery IJV and femoral vein CSA Calf volume	Inflight without cuffs Lower LVEDV and SV (vs preflight) CSA: IJV + 23% to +30%, Femoral vein: +33% to +70% Resistance: renal artery: −15% to −16%, Femoral artery −5% to −11% Inflights “with versus without” cuffs CSA: IJV −12% to −20%, Femoral vein +9% to +20% Increased resistance LBNP inflight/postflight versus pre-flight Less increase of femoral resistance and cerebral/femoral blood flow ratio Less increase in calf circumference
Jasien et al.^22^	ISS Multiple institutions and international partners and space agencies NASA, Houston Texas, Russian Federation State Research Centre Institute of Biomedical problems TP: NR D: 215 ± 72-day mission	To use multiple non-invasive methods (including IJV pressure) to assess intracranial pressure (ICP) before, during and after long-duration spaceflight, and to determine if use of LBNP could reduce ICP during spaceflight	Interventional Prospective cohort Repeated measurements -Pre-flight and post-flight postures: seated, supine, and 15° HDT -Inflight: D45 (FD45) and 150 (FD150) with and without LBNP -Postflight: R + 10, R + 30, R + 180	13 crewmembers (2 females, 11 males. Age (mean ± SD): 46 ± 6.6 Weight (mean ± SD): 81.5 ± 9.5 kg	Inflight versus pre-flight measurements of: - Cerebral and cochlear fluid pressure (CCFP) - Otoacoustic emissions (OAE) - Ultrasound measures of optic nerve sheath diameter (ONSD) - Ultrasound-based IJV pressure (IJVp) using VeinPress device Comparison of observed effects with modelled means (95% confidence intervals)	IJVP without LBNP Mean difference (95% CI) mmHg: - FD45 vs pre-flight supine: −2.6 (−6.6, 1.5) - FD45 vs pre-flight seated: 14.3 (10.1, 18.5) - FD45 versus 15° HDT: −4.1 (−0.1, −8.2) - FD45 vs FD150: −2.2 (−6.4, 2) IJVP with LBNP not measured
Lee et al.^23^	Lyndon B. Johnson Space Centre, NASA, Houston, TX, USA KBR, Houston, TX, USA Memorial Sloan Kettering Cancer Centre, NY, USA School of Kinesiology, University of Michigan, Ann Arbor, MI, USA Novespace, Bordeaux-Mérignac, F TP : 2018 D : 3 days	To characterise the relationships between gravitational level (Gz-level) and acute vascular changes	Interventional Prospective cohort Repeated measurements IJV CSA (and flow patterns), inferior vena cava (IVC) diameter, and common carotid artery (CCA) flow were measured using ultrasound in subjects while seated when exposed to 1.00-Gz, 0.75-Gz, 0.50-Gz, and 0.25-Gz during parabolic flight and while supine before flight (0-G analogue).	9 subjects (5 F, 4 M) Age: 39 ± 6 years old (mean ± SD; range: 34–50 years), Height: 171 ± 11 cm (157–187 cm) Weight 65 ± 10 kg (50–85 kg).	Right IJV CSA Doppler flow patterns IJV pressure Right CCA CSA and flow No issues Descriptive and graphical summaries Linear regression Generalised estimating equations using an independence correlation structure (GEE-Ind) to account for repeated measurements within subject Paired t-test	IJV area progressively increased from 12 (95% CI: 9–16) mm2 during 1.00-Gz seated to 24 (13–35), 34 (21–46), 68 (40–97), and 103 (75–131) mm2 during 0.75-Gz, 0.50-Gz, and 0.25-Gz seated and 1.00-Gz supine, respectively. IJV flow patterns shifted from the continuous forward flow observed during 1.00-Gz and 0.75-Gz seated to pulsatile flow during 0.50-Gz seated, 0.25-Gz seated, and 1.00-Gz supine. IJV pressure: minimal difference from 1.00-Gz supine to 0.25-Gz seated for two subjects and an increased IJV pressure in one subject. IJV pressure at 0.50-Gz seated (9.5 ± 3.4 mmHg) was lower than 1.00-Gz supine (19.1 ± 7.6 mmHg) for all five subjects [difference: 9.6 (95% CI: 5.0–14.1), *p* = 0.003]. Unable to detect differences in IVC diameter measured during 1.00-G seated and any level of partial gravity or during 1.00-Gz supine.
Marshall-Goebel et al.^2^ Multi-institution international fluid shifts study	KBR, Houston, TX. Institute of Biomedical Problems of the Russian Academy of Sciences, Moscow, Russian Federation University Hospital Trousseau, Tours, F Applied Biostatistics Laboratory, School of Nursing, University of Michigan, Ann Arbor. Department of Surgery, Henry Ford Hospital, Detroit, Michigan. Department of Orthopaedic Surgery, UC San Diego Medical Centre, University of California, San Diego NASA, Johnson Space Centre, Houston, TX TP: not explicit to protect the identities of participants D: Spaceflight 180 days	To assess IJV area and pressure, as well as characterise the Doppler flow velocity profile during spaceflight compared with various postures on Earth and to investigate if lower body negative pressure (−25 mmHg) is associated with reversing the headward fluid shift experienced during spaceflight.	Interventional Prospective cohort Design: longitudinal, with astronauts providing data from each of 3 postures preflight and postflight (seated, supine, head-down tilt), and 2 in-flight conditions observed at 2 periods (spaceflight and spaceflight with LBNP at approximate flight days 50 and 150) Non-invasive IJVP (mmHg) measured by compression US using the VeinPress device.	11 crew members Age (mean [SD]: 46.9 [6.3] years. Gender: 9 [82%] men. Body mass index (mean [SD] 26.4 [3])	Longitudinal study with comparison of spaceflight (+/− LBNP) at different times with pre-flight and postflight postures for analysis of: - Mean IJV area - Mean IJV pressure Analysis of - Flow patterns - Thrombus formation No issues in analysis Mixed models	Mean IJV area: -Pre-flight seated position 9.8 (95% CI, −1.2 to 20.7) mm^2^ -Spaceflight: 70.3 (95%CI, 59.3–81.2) mm^2^ (*P* < 0.001). Mean IJV pressure: -Preflight seated position: 5.1 (95% CI, 2.5–7.8) mm Hg -Spaceflight: 21.1 (95%CI, 18.5–23.7) mm Hg (*P* < 0.001). Stagnant (*n* = 5) or reverse (*n* = 1) flow in the IJV on approximate flight day 50 in 6 crew members (55%) Occlusive IJV thrombus (with stagnant flow): 1 crew member (same case as in Aunon S.M. et al (2020) publication Potential partial IJV thrombus (with stagnant flow): 1 subject retrospectively. LBNP −25 mmHg during spaceflight reduced IJV area and improved blood flow in 10 of 17 sessions (59%)
Martin et al.^24^	KBRwyle Science, Technology & Engineering Group, Houston, Texas MEI Technologies, Houston, Texas Universities Space Research Association, Houston, Texas NASA Johnson Space Centre, Houston, Texas TP: NR D: Four flights (Each flight lasted approximately 2.5 h)	To measure IJV pressure (IJVP) during normo- and hypo-gravity as an index of venous congestion. To determine whether IJVP, as a potential contributor to elevated ICP, was increased from normal (1 G) to weightlessness (0 G).	IJVP was measured during normal gravity (1 G - supine) and weightlessness (0 G - seated) produced by parabolic flight - at rest (end-expiration) and during controlled breathing manoeuvres (10 mmHg, 20 mmHg). Each flight included 40 parabolas, providing about 20 sec of 0 G or partial gravity per parabola. Each reduced gravity parabola was preceded and followed by a hypergravity phase of up to 1.8 G lasting about 20 sec. IJVP also was measured in two subjects during parabolas approximating Lunar (1/6 G) and Martian gravity (1/3 G). Non-invasive IJVP (mmHg) measured by compression US using the VeinPress device.	11 normal, healthy subjects (3 M, 8 F) Mean age: 39.5 years, range 27–60 yrs) Mean height: 168 cm, range: 157–196 cm; Mean weight: 66.6 kg, range: 50.5–109.9 kg)	Differences in IJVP between 1 G and 0 G tested using a paired T-test. No statistical issues A mixed-effects linear regression model to examine the effect of gravity and breathing manoeuvres on IJVP using dummy-coded grouping variables for gravity and pressure relative to 1 G seated. Bootstrap resampling performed to improve estimates of variance given the small sample size. Holm correction for multiple comparisons between baseline and each treatment condition.	IJVP was higher in 0 G than 1 G (23.9 ± 5.6 vs. 9.9 ± 5.1 mmHg, mean ± SD *P* < 0.001) in all subjects. IJVP increased as gravity levels decreased in two subjects. IJVP was greater in 0 G than 1 G at all expiration pressures (*P* < 0.01). IJVP is elevated during acute exposure to reduced gravity and may be elevated further by conditions that increase intrathoracic pressure.
Pavela et al.^25^	NASA, Houston, TX, USA. KBR, Houston, TX, USA. Department of Radiology, University of Texas MD Anderson Cancer Centre, Houston, TX, USA. Aegis Aerospace, Inc., Houston, TX, USA. TP: NR D: 2150 person-days spaceflight	To report the Doppler US findings of the bilateral IJV evaluations and discuss their relevance to a possible prothrombotic state	Interventional Prospective cohort Repeated measurements Duplex US of the bilateral IJV was conducted on all NASA astronauts terrestrially, and at three points during spaceflight. Respiratory manoeuvres were performed (normal-effort respiration, Valsalva, modified Mueller and manual compression of the contralateral IJV).	11 NASA astronauts (six male, five female)	Images were analysed for thrombosis, CSA and certain hemodynamic characteristics, including peak velocity (PV) and degree of echogenicity. Evaluation by matching terrestrial and in-flight ultrasounds. Right and left IJV CSA and IJV-PV measurements were compared with the Wilcoxon test. Separate mixed effects generalised linear models. Univariable linear regression models Univariate proportional odds logistic regression models	No thrombosis detected. IJV-CSA: The left IJV significantly smaller than the right IJV at terrestrial baseline and first and second in-flight exams (*p* = 0.04, 0.02, 0.01, 0.2). Compared to terrestrial baseline, the in-flight right IJV-CSA was significantly increased on all exams, and the left IJV-CSA was significantly increased on the third exam. Echogenicity: Six of 11 astronauts had mild-moderate echogenicity in the left IJV during spaceflight, but none had more than mild echogenicity in the right IJV. Peak velocity: Compared to terrestrial US, in-flight peak velocity was reduced and lowest in the left IJV. Two astronauts developed retrograde blood flow in the left IJV Respiratory manoeuvres Flow response as expected
Zamboni et al.^26^	Vascular Diseases Centre and Department of Physics and Earth Sciences, University of Ferrara, Ferrara, Italy Italian Space Agency European Space Agency (ESA) NASA TP: NR D: 6 months	To provide proof of concept for the feasibility of measuring IJV pulse variations before, during and after the experiment in microgravity. IJV pulse extrapolated from an US video recording of the IJV synchronised with ECG that produces a CSA time trace (IJV pulse trace)	Feasibility study A series of cross-sectional scans of bilateral IJVs lasting approximately 30 s on each side Six experimental sessions (the third and the fourth performed in the ISS).	A 37-y-old female astronaut	Mean and standard deviation of the IJV pulse waves and the phase relationship between such waves and P and T waves on the ECG calculated from approximately 30 cardiac cycles. Verification that such parameters had the same accuracy on Earth as under microgravity. Values in terms of sensitivity, specificity & accuracy but reference standard not reported. Many study limitations in the design, conduct, analysis and reporting.	The sensitivity, specificity and accuracy in microgravity did not significantly differ from those on Earth. On board the ISS, the parameters increased approximately by 15% in the first session, whereas in the second session, they decreased by more than 50%. Authors conclusion: “The experiment indicated the feasibility of deriving a IJV pulse trace from a B-mode US examination self-performed by an astronaut in microgravity”.

*Braslet* Bracelets, *CNES* Centre National d’Etudes Spatiales, *CO* cardiac output, *CSA* cross-sectional area, *CVP* central vein pressure, *D* day, *DVT* Deep venous thrombosis, *ECG* electrocardiogram, *ESA* European Space Agency, *HDT* Head down tilt, *ICP* intracranial pressure, *IJV* internal jugular vein, *ISS* International Space Station, *LBNP* Lower body negative pressure, *LVED* left ventricle end diastolic volume, *NA* Not applicable, *NASA* National Aeronautics and Space Administration, *NR* Not reported, *SF* spaceflight, *PD* prophylactic device, *SV* stroke volume, *UCSD* University of California San Diego, *US* Ultrasound.

Studies reported data on between 1 to 14 crewmembers. Most studies used repeated measurements and compared inflight with pre-flight and postflight measurements, and long-term with short-term inflight outcomes. The main measure assessed was the cross-sectional area (CSA) of the IJV, rarely the femoral vein. The other types of evaluation included Doppler flow patterns, echogenicity, peak velocity, vein pressure and IJV pulse, performed in some studies with, and without venous interventions (Table 3).

### Methodological quality of included studies

There was a substantial variability between studies in year of publication, study design, objectives, outcomes, V-US method, quality, statistical analyses, and reporting. Reporting issues were found mainly in older studies. Between-study heterogeneity prevented from performing meta-analysis.

### V-US changes induced by microgravity in spaceflight studies

The purpose was to identify changes induced by microgravity in the upper body venous system to account for when carrying out explorations in spaceflight. Of the 16 spaceflight studies included, 13 focused solely on microgravity-related changes^14–24,26,28^, one exclusively on DVT detection^1^, and two studies on both microgravity-related changes and DVT detection^2,25^. Most studies reported inflight V-US changes in contrast with terrestrial findings (pre-flight mainly) at the level of the IJVs, rarely the femoral veins. Most changes were reported as an increase in IJV (and femoral vein) CSA and volume, and in IJV pressure, associated to a decreased blood flow velocity, increased blood echogenicity most frequently seen in the left IJV, and slow, retrograde left IJV blood flow. Results of left IJV investigation reported were different and need be analysed separately from those obtained at the right IJV (Supplementary Table 6). A summary of the reported findings is displayed in Table 4.

**Table 4:** Summary of in-flight venous ultrasound changes.

Venous US assessment	Findings
Peripheral vein morphology	Increase of the IJV CSA from FD1 (flight day one)^28^ and the IJV volume^16^ during spaceflights.
	Right IJV CSA significantly different between in-flight values and several angles of the Russian tilt-table pre-flight protocol (CSA values were higher with negative angles and vice versa), except for the 0° measurement^17^.
	Increase of the femoral vein section from FD9^28^ as opposed to reduced femoral vein section during head-down tilt (HDT)^14^, and decrease of calf and gastrocnemius veins^15^.
	In-flight, jugular and femoral veins increased from week 1 to month 5–6^14,18^, with a significant difference in femoral area between months 1 and 6^18^.
	Results regarding IJV distensibility are controversial and were reported as both reduced^28^ or unchanged^18^.
Peripheral vein pressure	In-flight on FD45 reported not to be different from pre-flight values in supine position, but to be higher than pre-flight in a seated position and lower than pre-flight at 15° HDT, and not different from FD150^22^.
	IJV area and pressure reported to be increased with chronic microgravity exposure^2^ and with partial gravity acute exposure^23^.
	IJV pressure reported to be increased as gravity levels decreased and to be greater in 0 G than in 1 G at all expiration pressures^24^.
IJV flow patterns	Reported under acute hypogravity exposure as a function of subject position and the level of gravity^23^.
	An IJV blood flow velocity grading scale was reported in microgravity^2^.
	A decreased peak velocity in both IJV was found to be associated with increased IJV CSA, increased blood echogenicity most frequently seen in the left IJV, and slow, retrograde left IJV blood flow reported in two of 11 astronauts^25^.
IJV pulse trace parameters	Values of IJV pulse trace parameters in a single female astronaut reported^26^.

*US* Ultrasound, *IJV* Internal jugular vein, *CSA* Cross-sectional area, *FD* Flight Day, *HDT* Head down tilt.

### Reversal of V-US changes with venous interventions in-flight

The objective was to identify in case of venous congestion in the IJV what type of intervention was able to reverse the effect of microgravity and restore optimal conditions (i.e., normal terrestrial venous morphological and haemodynamic aspects (1 G supine)) in-flight (Table 5 and Supplementary Table 7), thus determining that venous congestion was likely related to microgravity and not to DVT. Among 16 included studies, 10 reported on such interventions. All used quantitative data analyses with continuous variables. None have reported results as categorical data that could help analyse the sensitivity of V-US to changes induced by each intervention.

**Table 5:** Summary of the effect of in-flight venous interventions.

Venous interventions	Findings
LBNP	Application of LBNP (−25 mmHg and −45 mmHg of 10 min with a transition step of −35 mmHg): The calf volume increased as the pressure decreased (Arbeille et al.)^28^.
	Application of LBNP −25 mmHg restored variables at least back to pre-flight supine levels. From reported figure: IJV volume returns to pre-flight supine level in 10/12 astronauts (Arbeille et al.)^16^.
	LBNP −25 mmHg reduced IJV area and improved blood flow (Marshall-Goebel et al.)^2^.
	LBNP −45 mmHg: Less increase of calf circumference, femoral resistance, and cerebral/femoral blood flow ratio inflight/postflight than pre-flight (Herault et al.)^21^.
Cuffs (Bracelets)	With versus without cuffs: decreased jugular area, increased femoral area (Herault et al.)^21^.
	Reduction of cephalic congestion and jugular vein area, no noticeable effect on cerebral blood flow, femoral vein area higher (Fomina et al.)^19^.
	Increased femoral vein area, decreased IJV area (Hamilton et al.)^20^.
Respiratory manoeuvres	Increased femoral vein area with bracelets, little additional increase with bracelets + Valsalva (Hamilton et al.)^20^.
	Increased femoral vein with Valsalva (Hamilton et al.)^20^.
	IJV pressure appears to increase as the level of gravity decreases (Martin et al.)^24^.
	Decreased IJV area with bracelets, Mueller and both bracelets + Mueller (Hamilton et al.)^20^.
	Flow induced by Mueller manoeuvre (Auñón-Chancellor et al.)^1^.
	Modified Mueller increased peak velocity and reduced echogencity (Pavela et al.)^25^.
	Modified Mueller and contralateral compression reversed the flow direction to antegrade (Pavela et al.)^25^.

#### LBNP

The application of LBNP at −25 mmHg for 30 min was able to restore variables at least back to pre-flight supine levels and reduced the associated flow and tissue disturbances^16^. The calf volume increased as the LBNP negative pressure decreased (−25 mmHg and −45 mmHg of 10 min with a transition step of −35 mmHg)^28^. There was less increase in calf circumference at LBNP −45 mmHg^21^. This was associated with a reduction of CSA and an improvement of blood flow in the IJV. The IJV volume returns to pre-flight supine level in 10/ 12 astronauts (83%) from our analysis of reported figure^16^.

#### Cuffs (Bracelets)

With versus without cuffs, IJV area decreased, and femoral vein area increased^19–21^. Cephalic congestion was reduced without a noticeable effect on cerebral blood flow^19^. As cited by Arbeille^15^, a 30 mmHg thigh-cuff pressure positioned at the upper part of the thigh traps blood and other fluids in the superficial leg veins and tissues, and consequently, reduces the IJV area in-flight, restoring it to pre-flight or pre-bed rest levels^21,29^.

#### Respiratory manoeuvres

An increase in femoral vein area was found with the Valsalva manoeuvre, with a little additional increase when adding Valsalva manoeuvre to bracelets as compared to bracelets alone^20^. Internal jugular vein area decreased with bracelets, with the Mueller manoeuvre and with the combination of both bracelets + Mueller manoeuvre^20^. Venous flow was found to be induced by the Mueller manoeuvre^1^. A modified Mueller manoeuvre increased peak venous flow velocity and reduced echogenicity^25^. In two individuals with retrograde blood flow, both the modified Mueller manoeuvre and the contralateral manual compression of the right IJV reversed flow direction to antegrade while the manoeuvre was performed^25^.

In total, venous interventions were on average effective in reducing or even normalising in-flight changes (Table 2 and Supplementary Table 9). Limited data was available on individually induced effect of interventions.

### Appropriateness of spaceflight ultrasound for venous assessment and DVT detection

The objective was to capture and highlight the gaps and technical issues associated with performing spaceflight V-US of the neck/ upper limb venous system in comparison with optimal methods on Earth.

Systematic reviews of diagnostic accuracy studies^30–32^ and diagnostic management^33^ studies in patients with clinically suspected upper extremity DVT on Earth were reviewed. They show a high diagnostic performance of V-US testing. In the systematic review of Patel et al.^32^, the pooled estimates for duplex US sensitivity and specificity from 7 studies^34–40^ were 87% (95% CI, 73–94) and 85% (95% CI, 72–93), respectively^32^. The diagnostic performance seems lower than it should actually be due to the inclusion of screening of asymptomatic catheter-induced subclavian vein thrombosis studies in the meta-analysis^38^. Sensitivity is lower in the screening setting as the thrombus is usually small and non-occlusive^41^. In a single-centre prospective diagnostic management study, among 337 symptomatic outpatients in whom the diagnosis of upper extremity DVT was ruled-out by colour-Doppler ultrasound and anticoagulant treatment was withheld, only one patient presented a DVT event during a 3-month follow-up resulting in a failure rate of 0.30% (95% CI, 0.05–1.68%)^33^. A normal upper extremity V-US finding based on duplex ultrasound (with or without colour Doppler ultrasound) can safely exclude DVT.

As stated above, appropriateness criteria for an optimal V-US testing on Earth were set-up after review of studies and expert panel discussions (Table 6), as follows:

1. For crew performing the V-US to be comparable to terrestrial environments, the study should have included a qualified physician astronaut or sonographer on board, or an astronaut with ground-based manual remote control (i.e., tele-operated echograph and motorised probe), both with a specialised interpretation/ analysis of US images. Crew was considered not comparable to terrestrial one if there was only ground-based remote guidance (by voice), even if the astronaut was adequately trained in positioning the probe and capturing images and if the US images were subject to specialist interpretation/analysis. The ©2021 and ©2023 IAC (Intersocietal Accreditation Commission) Standards and Guidelines for Vascular Testing Accreditation (Published November 15, 2021, and June 1, 2023) consider training and experience in venous duplex ultrasound adequate for established practice for a physician who has worked in a vascular facility for at least the past three years and has interpreted 300 diagnostic cases. In comparison, the astronauts have before their flight a familiarisation session (approx. 3 h). The appropriate V-US method for “upper extremity” DVT screening in space is more complex than V-US limited to the IJV or the lower-limb peripheral veins and requires much more extensive training/ expertise. Although limited compression ultrasound of peripheral veins can be performed accurately by an operator with little training as shown in terrestrial studies in symptomatic patients^42,43^, there is a significant heterogeneity between studies in terms of the degree of training required^44^. The context of UEDVT screening during spaceflight is different as astronauts are asymptomatic and therefore the thrombus may be small and non-occlusive^45,46^, and the location may concern isolated central (i.e., “intrathoracic”) or deep IJV as in the reported thrombosis case^1,2^. Remote (manual) control has many advantages^12,47^. It allows full control of the probe orientation by the expert and provides diagnoses in 97% of cases^48^. Remote guidance is highly dependent not only on remote guider expertise and instructions but also on individual operator skill to self-scan and prevent errors from probe/hand movement. Remote guidance is sufficient for superficial vessel examinations but not suited for deep or superficial organs^48^. A recent publication using 3D scan on same structure found more consistent results with the motorised probe compared with remote guidance^49^.
2. For in-flight ultrasound equipment to be appropriate and optimal for venous assessment and be comparable to terrestrial standards, it should have included equipment with high resolution imaging devices/ scanners, various ultrasound emission frequencies in B-mode (e.g., L12-5 MHz for peripheral veins, P5-2 MHz for intrathoracic veins) and adequate transducer sizes (small transducers for central veins) integrating different US modalities (B-mode + colour-flow + doppler ultrasound). Otherwise, in-flight equipment was considered to have limitations and to be inappropriate for an optimal V-US assessment.
3. For in-flight assessment sites to be comparable to terrestrial ones, both peripheral deep and superficial veins of the arm, the axilla, and the neck, and central (i.e., intrathoracic) veins should have been examined for DVT detection and thus assessment should have included not only jugular veins but also other peripheral and accessible intrathoracic veins of the neck/ upper limb system (subclavian vein and innominate trunk). This criterion was applicable only to studies involving DVT detection.
4. For in-flight V-US assessment method to be comparable to terrestrial one, it should have included both morphological and hemodynamic assessment modalities. The high performance of V-US examination in terrestrial studies has been achieved by combining B-mode ultrasound and Doppler (i.e., colour Doppler ultrasound, duplex ultrasound). Because central veins cannot be systematically and adequately examined due to the presence of overlying bones, imaging and doppler patterns analyses should be combined. This criterion was applicable only to studies involving DVT detection.
5. For V-US DVT detection criteria in-flight to be comparable to terrestrial one, the diagnosis should have relied on the direct visualisation of the thrombus on B-mode or colour-Doppler ultrasound and on vein incompressibility (only for peripheral veins), and abnormal flow patterns within or distal to the thrombosis on duplex ultrasound. The combination of direct and indirect identification of vein thrombosis is essential. Indirect investigation of the central veins based on the Doppler signal^50^ is a key element for the diagnosis exclusion of central (intrathoracic) DVT when it shows normal phasicity with respiration and cardiac cycle at the level of the axillary vein (or subclavian and brachiocephalic veins) in supine position^32,51,52^. Loss of phasicity is diagnostic of central vein obstruction^32,51,52^ in the absence of extrinsic compression. When several parameters are evaluated in combination (thrombus visualisation, absence of spontaneous flow, absence of phasicity with respiration/ cardiac cycle, vein incompressibility), venous ultrasound is a reliable method for DVT detection^30,39,53^.

**Table 6:** Appropriateness criteria and comparison of inflight venous ultrasound with terrestrial standards.

Appropriateness criteria	DVT studies (*N* = 3)	Other studies (*N* = 15)
Appropriate crew for venous ultrasound assessment (experience)	Experts: 0	Experts: 4
- Expert physician or sonographer on board OR	Remote control: 0	Remote control: 0
- Ground-based manual remote control	NR: 0	NR: 4
Appropriate in-flight equipment		
- Peripheral veins: High-frequency emission probe in B-mode US (e.g., L12-5 MHz) – Colour-Doppler US – Duplex US	Yes: 3 (No: 0, NR: 0)	Yes: 7 (No: 2, NR: 6)
- Central veins: Low- frequency emission probe in B-mode US (e.g., C5-2 or P5-2 MHz) - Colour-Doppler US - Duplex US (with a small size probe for intrathoracic venous assessment)	Yes: 0 (No: 3, NR: 0)	Yes: 4 (NA: 10, NR: 1)
Appropriate assessment sites (both to be combined for venous thrombosis detection)	Yes: 0	Adapted to the study
- Peripheral veins: Whole venous network AND	- Only IJV assessed	
- Central veins: Direct assessment (subclavian vein and innominate trunk) + Indirect assessment from peripheral veins	- No direct assessment of central veins	
Appropriate assessment methods (both to be combined for venous thrombosis detection)	Yes: 3 (Both assessment methods combined)	Adapted to the study
- Morphological assessment: echogenicity, flow direction, thrombosis image, compression US test (for peripheral veins) AND		
- Hemodynamic assessment (Colour-Doppler US + Duplex US): filling of the vein segment, blood flow velocity (doppler pattern, magnitude, direction)		
Appropriate criteria to rule-out venous thrombosis:		
- Peripheral veins: vein compressibility (B-mode) + absence of thrombus direct image (B-mode) + normal filling of the vein segment (Colour-Doppler)	Yes: 3	NA
- Central veins: absence of direct thrombus image and absence of vein obstruction (B-mode and Colour-Doppler) + normal phasicity with respiration and cardiac cycle at the level of the axillary vein (in supine position on-Earth) for the assessment of intrathoracic veins (using duplex ultrasound)*	Not assessed.	
Appropriate criteria to rule-in venous thrombosis:		
- Peripheral veins: non-full compressibility of the vein segment (B-mode) + partial or complete venous obstruction (B-mode and Colour-Doppler) + high quality thrombosis direct image (B-mode)	Main issue: Quality of thrombosis image.	NA
- Central veins: partial or complete venous obstruction (B-mode and Colour-Doppler) + high quality thrombosis direct image (B-mode) + loss of phasicity with respiration and cardiac cycle at the level of the axillary veins in supine position for the assessment of intrathoracic veins (using duplex ultrasound)	Not assessed.	

Among the 16 included publications: one reported exclusively on DVT study, two on both DVT studies and other types of studies, and 13 exclusively on other types of studies. Other studies = Not DVT studies. Neck and upper extremity peripheral venous segments: internal jugular veins (IJV), brachial and axillary veins – Neck and upper extremity central venous segments: deep IJV segment, subclavian, brachiocephalic venous trunk, superior vena cava. Remote Control is Remote Manual Control.

*Yes* appropriate, *No* not appropriate, *NA* not applicable, *NR* not reported.

^*^+ reversal of stasis phenomenon in-flight under venous interventions (i.e., mainly modified Mueller manoeuvre and the contralateral manual compression of the right IJV).

Studies in space can be divided into DVT and non-DVT studies. In DVT studies^1,2,25^, one case of ultrasound-detected DVT was reported in two separate papers in an asymptomatic astronaut with description of vascular aspects at diagnosis^1,2^ and follow-up^1^. A second case was reported based on retrospective analysis of ultrasound images in another asymptomatic astronaut and adjudicated as a “likely” DVT^2^. In another study intending to screen for DVT, all astronauts (*n* = 11) remained asymptomatic and no DVT was detected by US^25^. All three studies assessed morphological and flow patterns with, and without venous interventions^1,2,25^.

Tables 6 and 7 summarise appropriateness of spaceflight V-US with terrestrial V-US standards relevant to the crew performing V-US, equipment, assessment sites, assessment methods, and DVT detection criteria as follows:

1. For crew performing V-US: In-flight V-US was performed by expert on board physician astronaut or sonographer in four publications (Tables 6 and 7). None of these was involving DVT screening. Indeed, in the two DVT publications reporting on the same thrombosis case^1,2^, there was a physician astronaut on board likely familiar with vascular ultrasound but her/ his extent of training and expertise in V-US for UEDVT screening is unknown. No publication reported V-US performed under ground-based remote manual control, four publications including a third DVT study^25^ reported V-US performed under remote verbal guidance, and there was a reporting issue regarding the crew in six other publications. Overall, crew performing in-flight US was only in four publications^18,19,23,24^ comparable to the terrestrial clinical standard (Tables 6 and 7 and Supplementary Table 8).
2. For V-US equipment: In the three DVT studies, the emission frequency was too high and the probe too large (linear) to enable investigating IJV deep segment, subclavian and intrathoracic veins which may bias the V-US results. In the other studies, V-US equipment was appropriate and adapted to the study objective (Tables 6 and 7 and Supplementary Table 9).
3. For V-US assessment sites: Assessment of upper extremity venous system was limited to the IJV according to the objective of the studies. In DVT studies, the other peripheral veins and intrathoracic veins were not directly evaluated as in terrestrial DVT screening on Earth (Tables 6 and 7 and Supplementary Table 10).
4. For V-US assessment methods: In the DVT studies, V-US methodology was comparable to terrestrial standards as morphological and hemodynamic assessments were combined. In the other studies, V-US methodology was adapted to the purpose of the study, mainly the assessment of cephalad congestion on B-mode US (dilation, increased echogenicity, increased vein pressure) and Doppler flow characteristics (magnitude, direction, aspects, phasicity) (Tables 6 and 7 and Supplementary Table 11).
5. For DVT diagnostic criteria : The diagnosed left IJV thrombus reported in two separate papers^1,2^ was described as echogenic and occlusive with the vein almost compressible and associated with stasis (spontaneous echo-contrast), absence of flow and flow reversal. Images of thinned thrombus was obtained on follow-up V-US during spaceflight. Venous flow returned to normal upon landing on Earth. As the diagnostic issue was in the deep proximal segment of IJV where compression US test is difficult to acquire, the diagnosis was based not only on the absence of venous flow which may be due to congestion phenomenon as well, but on its combination with the direct image of the thrombus. Unfortunately, no video recordings were available to enable discriminating between a stasis image and a true direct thrombus image. The quality of the reported IJV thrombus image although acquired with a L12-5 MHz probe on oblique sagittal planes is not sufficient to indicate a thrombosis within the images of stasis. No comparative diameter (or volume) measurements were performed on follow-up to attest of the potential thrombus evolution. The reported thrombus consistency pattern may depend on V-US compression force, and the volume reduction estimate on probe positioning. LBNP in-flight and HDT postflight countermeasures were not performed for this astronaut. The increase in flow following the Mueller manoeuvre^54,55^ and the absence of altered flow patterns post-flight are more suggestive of microgravity-related stasis (Tables 6 and 7). The second DVT case following a retrospective analysis of ultrasound images from astronauts was reported as a “potential thrombosis” and was described as a partial IJV thrombus^2^. In the surveillance DVT study^25^, a bilateral IJV screening was performed and assessed morphological and flow patterns in 11 astronauts. Despite the absence of any thrombosis, vein congestion and abnormal flow characteristics (such as reduced peak velocity, “mild–moderate” echogenicity in six participants, and retrograde blood flow in two participants) were observed and were prominent in the left IJV^25^. This suggests a lack of specificity of venous congestion and abnormal flow patterns criteria in DVT screening during spaceflight and the need of venous interventions to restore normal diameter and flow patterns as was done in this study^25^.

**Table 7:** Bias assessment in spaceflight venous ultrasound in comparison with terrestrial standards.

Author	Crew performing V-US	Equipment	Venous US sites	V-US assessment method	Venous thrombosis criteria
Arbeille et al.^28^	Reporting issues	Reporting issues (Old study published in 1994 at a conference meeting)	Assessment limited to IJV and femoral vein for a different purpose	Morphology: Vein area	Not applicable
Arbeille et al.^14^	Reporting issues	Summary of multiple records published in 1988, 1992, 1994, 1996, 1997, 1998, 1999 and 2000	Assessment limited to IJV and femoral vein for a different purpose	Morphology: Vein area/ volume	Not applicable
Arbeille et al.^15^	Remote guidance	Reporting issues. Only B-mode US used	Assessment limited to IJV and femoral veins (and other LL vein segments) for a different purpose	Morphology: Vein area	Not applicable
Arbeille et al.^16^	Remote guidance	Reporting issues	Assessment limited to IJV (and middle cerebral vein) for a different purpose	Morphology and haemodynamics	Not applicable
Auñón-Chancellor et al.^1^	Physician astronaut (extent in training in V-US for DVT screening unknown) Remote guidance	For DVT detection: Need for lower US emission frequency and smaller US probes to examine deeper vein segments (no other limitation)	Assessment limited to IJV. No direct assessment of intrathoracic veins	Morphology and haemodynamics	Uncertainty regarding direct thrombosis image in the IJV. Image more compatible with venous stasis (see comments in text)
David et al.^17^	Reporting issues	Only B-mode US used	Assessment limited to IJV for a different purpose	Morphology: Vein area	Not applicable
Fomina et al.^18^	Reporting issues on interpretation of US but US performed by physician	Old studies and reporting issues (Summary and analysis)	Assessment limited to IJV and femoral vein for a different purpose	Morphology: Vein area	Not applicable
Fomina et al.^19^	Reporting issues on interpretation of US but US performed by physician	Old study and reporting issues	Assessment limited to IJV and femoral vein for a different purpose	Morphology: Vein area	Not applicable
Hamilton et al.^20^	Remote guidance	Only B-mode US used	Assessment limited to IJV and femoral vein for a different purpose	Morphology: Vein area	Not applicable
Herault et al.^21^	Reporting issues	Old study, old equipment (5 Mhz probe used for IJV and femoral vein studies)	Assessment limited to IJV and femoral vein for a different purpose	Morphology: Vein area	Not applicable
Jasien et al.^22^	Reporting issues	No limitation. Different equipment for a different purpose: Pressure measurement using US	Assessment limited to IJV for a different purpose	Morphology and haemodynamics: Vein pressure measurements	Not applicable
Lee et al.^23^	No limitation US performed by sonographer	No limitation Different equipment for a different purpose	Assessment limited to IJV vein for a different purpose	Morphology and haemodynamics: Vein area and vein pressure measurements	Not applicable
Marshall-Goebel et al.^2^	Physician astronaut (extent in training in V-US for DVT screening unknown) Remote guidance	For DVT detection: Need for lower US emission frequency and smaller US probes to examine deeper vein segments (Otherwise no limitation)	Assessment limited to IJV No direct assessment of intrathoracic veins	Morphology and haemodynamics	Same case of IJV thrombosis as in Auñón-Chancellor, S.M. et al. report (same comments). Second “potential thrombosis” on retrospective data analysis.
Martin et al.^24^	No limitation US performed by sonographer	No limitation Different equipment for a different purpose	Assessment limited to IJV for a different purpose	Morphology and haemodynamics: Vein pressure measurements	Not applicable
Pavela et al.^25^	Remote guidance	For DVT detection: Need for lower US emission frequency and smaller US probes to examine deeper vein segments (Otherwise no limitation)	Assessment limited to IJV. No direct assessment of intrathoracic veins	Morphology and haemodynamics	No DVT detected
Zamboni et al.^26^	Reporting issues	Reporting issues Different purpose (assessment of IJV pulse)	Assessment limited to IJV for a different purpose	Morphology and haemodynamics: IJV pulse trace parameters	Not applicable

In total, in the DVT studies (*n* = 3), only the V-US assessment modality criterion was fully appropriate. Crew performing V-US, used equipment, measurement sites and DVT diagnosis criteria were not entirely comparable to optimal terrestrial clinical practice. In the other studies (*n* = 15), all criteria (equipment, measurement sites and US modalities) were appropriate for the study objective except the crew experience criterion, which was appropriate in only four studies.

## Discussion

**Fig. 2:**
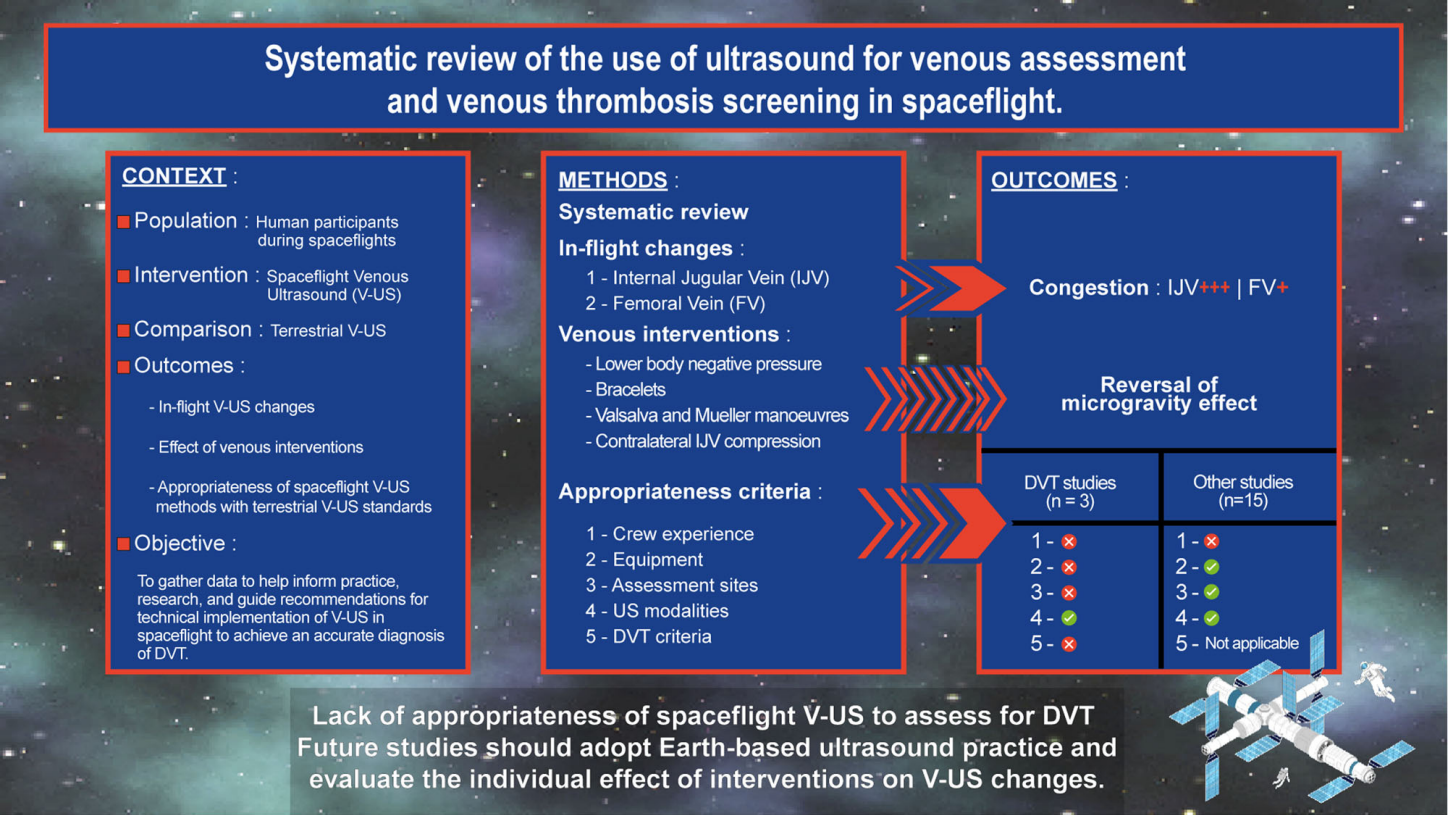
Summary of study results in spaceflight. Context, methods and findings regarding microgravity-related changes and effect of venous interventions to reverse them, and appropriateness of spaceflight venous ultrasound with terrestrial standards.

To the best of our knowledge, this is the first review investigating knowledge gaps between spaceflight and on-Earth V-US assessment. Results are summarised and shown in Fig. 2. Two types of barriers to optimal V-US evaluation were identified. They were either related to changes induced by microgravity or related to testing. Taking these results into account will help to make the necessary changes in the practice of V-US exploration in spaceflight and improve its diagnostic performance for the detection of DVT in the upper body venous system.

A systematic review combining spaceflight and ground-based analogue data on the effect of microgravity on the human venous system^3^ reported similar findings/ changes related to cephalad venous congestion with increased vein dilation and venous pressures and decreased/ reversed flow in microgravity. Previously published studies and reviews^56–58^ about interventions have assessed the role of in-flight interventions to prevent pathological and vascular changes but not as tools to improve diagnostic performance as in our review.

This review was restricted to spaceflight and did not include ground-based analogue studies. On the ground, V-US assessment is performed by highly specialised experts as opposed to in-flight V-US assessment performed by rather inexperienced crewmembers. According to Arbeille et al.^14^, IJV size changes differ between HDT and spaceflight probably due to differences between the intensity of the forces causing the fluid shift, and overall differences in mechanisms between the two conditions^17^.

A risk of publication bias cannot be excluded. The Cochrane handbook recognises that it is possible studies may be missing from a review^59^. As stated in the methods section, we relied upon a comprehensive search strategy to ensure that as many relevant studies are included as possible by searching grey literature, by including all studies regardless of their publication language (four studies published in Cyrillic were translated by professional scientific translators into English), and by contacting authors.

There is a risk of major reliability issues (repeatability and replicability of results) within studies in relation to the measurements of venous CSA and volume, Doppler flow characteristics and venous pressure. These measurements are not entirely precise even on Earth and when performed by experienced sonographers because of potential variability of explored venous segment, and variability of vein diameter and Doppler signal during cardiac cycle and respiration (and posture on Earth). As for venous pressure, it depends on the adequate and exact amount of compression needed to collapse the vein by the operator.

Conduct, analysis and reporting of methods and study results revealed significant shortcomings that must be addressed in future in-flight DVT studies. Reporting issues were related to the conduct of V-US (staff, training, remote control, annotation procedure, image captures, interpretation), the equipment used (Supplementary Table 9), V-US findings (Supplementary Table 6), assessment sites (Supplementary Table 10) and V-US methods (Supplementary Table 11). Most of the studies have included small sample sizes that may have biased the results. The sample size of spaceflight research is often small due to the time and financial cost of training astronauts^60,61^.

Our review has some limitations. Our data synthesis is only qualitative. Between-study heterogeneity and potential lack of reliability of measurements prevented from performing a quantitative synthesis (which was not in the scope of the objective). Extreme levels of heterogeneity in the use of outcome measures and a lack of study replication prevent the implementation of gold-standard meta-analysis techniques^62^. This, in addition to methodological issues within the included studies (lack of consistency in data collection within repeated measures studies^63,64^, lack of controlled trials), would have resulted in a lack of accuracy of measurements in our review and prevented us from any further quantitative assessment. Overall, the lack of quantitative synthesis should not have an impact on our objective. It was not possible to assess clinical probability due to the very small number of patients assessed and cases of venous thrombosis reported, and simply because patients were asymptomatic. In the context of spaceflight, clinical prediction rules need to be developed and validated considering the symptoms and signs related to the congestion phenomenon and based on a valid diagnostic outcome before they can be proposed and used in practice.

There are several implications for practice during spaceflight:

1. The present study revealed that current ultrasound screening tests for DVT in-flight need further development. It is essential to account for the various possible biases encountered in the present review when performing V-US in space.
2. Venous Ultrasound assessment “requires a great deal of training to perform ultrasound examinations, which can be difficult and time consuming, especially if the astronaut does not have a medical background”^12^. The aim of the studies carried out during spaceflights was to assess changes associated with microgravity and not to screen for DVT. Reported IJV thrombosis in spaceflight was unexpected, and astronaut were not familiarised with this type of testing. Assessing microgravity-related changes necessitated the use of sections mostly limited to the IJV. The search for “upper extremity” DVT in space is exposed to much greater technical difficulties, not so much for the assessment of peripheral veins where the analogy with the veins of the lower limbs is possible with interesting results and little training^42–44^, but above all for the exploration of central/“intrathoracic” veins (i.e., deep proximal IJV, subclavian vein, brachiocephalic vein, etc.). Exploration is no longer carried out at 2 or 3 points or based on the compression test but based on the sonographer’s ability to perform a complete assessment of the entire venous network, both directly (i.e., morphologically) with B-mode and colour doppler US and indirectly (i.e., haemodynamically) by analysing blood flow with duplex US. Two additional difficulties can be added to V-US assessment in space: the likely predominance of isolated anomalies in central veins and the challenge to distinguish the thrombus image from the very common stasis image, hence the importance of using dynamic manoeuvres to eliminate the stasis effect.
3. Because astronaut training is not sufficient to reduce the lack of reliability of V-US assessment, it is essential to adopt different training standards and to shift to the use of other systems such as tele-operated US systems^12^ with motorised probes (or maybe farther in the future, AI-based systems or systems using augmented reality).
4. It is important to be aware of the risk of over-diagnosis of DVT in the upper body venous system in space. A comprehensive investigation of the whole venous network (peripheral and central veins) is essential to identify an image of a real thrombosis and to clearly distinguish thrombosis from venous congestion. The use of venous interventions during spaceflights may be helpful to re-establish terrestrial conditions for a better V-US assessment.

There are also many implications for research and medical space operations:

1. Our results will provide a useful roadmap of desired diagnostic ultrasound methods and criteria that could be assessed and validated in future studies allowing set up of space-dedicated diagnostic algorithms facilitating decision-making. Moreover, the present results may help in designing studies on crew training and standardised image interpretation criteria. However, unexpected findings might occur with higher probability during a thorough research protocol.
2. In spaceflight, the diagnostic accuracy of terrestrial US criteria is unknown. Slow, stagnant, retrograde blood flow, especially in the IJV, due to volume overload and elevated venous pressures secondary to the classic cephalic fluid shift^8,56,65^, may mimic the aspects encountered in venous thrombosis. It is important to improve V-US scanning conditions by decreasing central venous pressure and venous congestion through the effect of venous interventions such as thigh cuffs, LBNP, Mueller manoeuvres and contralateral IJV compression. This could restore a venous flow with normal respiratory and cardiac phasicity useful for the investigation of intrathoracic veins, and restore a normal vein size that could facilitate the compressibility of a thrombosis-free peripheral vein segment and thus improve diagnostic specificity in-flight. This needs to be addressed in a study with adequate sample size that could assess the ability of interventions (and their intensity levels) on restoring normal blood flow and normal vein sizes to facilitate US-based DVT screening.
3. More effort needs to be put into developing new diagnostic technologies and maturing emerging terrestrial technologies for use in space to individualise thrombosis direct image at the level of the peripheral veins and the central veins. Equipment should be adapted to the context of missions beyond ISS. Ultrasound equipment for Artemis mission (hardware, motorised probe), not yet selected, will be by necessity a hand-held unit. It is not impossible for a hand-held unit to have many (or all?) of the functionality of the laptop-sized units that are currently on ISS. Time lags during sessions will increase as we move beyond ISS. It is remote guiding, not robotic probe operation, that will break first. Increasing the up-commanding lags does not necessarily prevent operation of the robotic probe.
4. Finally, it is highly important to set-up reporting methods specifically tailored for studies performed in space.

This systematic review shows that spaceflight V-US studies carried out to date are not of sufficient quality to assess for DVT, due to microgravity-related changes and testing methodology used. Findings from this systematic review will help inform practice and research towards optimal conditions of use adapted to a spaceflight situation. Future studies should adopt Earth-based ultrasound practice and evaluate the individual effect of interventions on V-US morphological and hemodynamic changes.

### Supplementary information

SUPPLEMENTARY INFORMATION

Reporting Summary

## Data Availability

All data relevant to the study are included in the article or uploaded as online supplementary information (see supplementary information). No code or other access needed.
