# Author Correction: Systematic review of the use of ultrasound for venous assessment and venous thrombosis screening in spaceflight

**DOI:** 10.1038/s41526-024-00362-y

**Published:** 2024-02-16

**Authors:** Antoine Elias, Tobias Weber, David A. Green, Katie M. Harris, Jonathan M. Laws, Danielle K. Greaves, David S. Kim, Lucia Mazzolai-Duchosal, Lara Roberts, Lonnie G. Petersen, Ulrich Limper, Andrej Bergauer, Michael Elias, Andrew Winnard, Nandu Goswami

**Affiliations:** 1 Cardiology and Vascular Medicine, Sainte Musse Hospital, Toulon Hospital Centre, Toulon, France; 2 Clinical Research and Innovation, Sainte Musse Hospital, Toulon Hospital Centre, Toulon, France; 3 Investigation Network On Venous Thrombo-Embolism | French Clinical Research Infrastructure Network (INNOVTE | F-CRIN), Toulon, France; 4 Space Medicine Team (HRE-OM), European Astronaut Center (EAC), European Space Agency (ESA), Cologne, Germany; 5 KBR, Cologne, Germany; 6 Centre of Human and Applied Physiological Sciences, King’s College London, London, UK; 7 Faculty of Medicine, Memorial University of Newfoundland, St. John’s, NL Canada; 8 University of Northumbria at Newcastle, Newcaslte-upon-Tyne, UK; 9 Space Biomedicine Systematic Review Methods Group, Wylam, UK; 10 Faculty of Health, University of Waterloo, Waterloo, ON Canada; 11 Department of Emergency Medicine, Faculty of Medicine, University of British Columbia, Vancouver, BC Canada; 12 Department of Angiology, Lausanne University, Lausanne, Switzerland; 13 King’s Thrombosis Centre, Department of Haematological Medicine, King’s College Hospital NHS Foundation Trust, London, UK; 14 Institute of Pharmaceutical Sciences, King’s College London, London, UK; 15 Department of Aeronautics and Astronautics, Massachusetts Institute of Technology, Cambridge, MA USA; 16 German Aerospace Center (DLR), Institute of Aerospace Medicine, Cologne, Germany; 17 University of Witten / Herdecke, Department of Anaesthesiology and Critical Care Medicine, Merheim Medical Center, Hospitals of Cologne, Cologne, Germany; 18 Department of Surgery, LKH Südsteiermark, Wagna, Austria; 19 Gravitational Physiology and Medicine Research Unit, Division of Physiology, Otto Loewi Research Center, Medical University of Graz, Graz, Austria; 20 Critical Care Medicine, St. Vincent’s Medical Center, Hartford Healthcare, Bridgeport, CT USA; 21 The Frank H. Netter MD School of Medicine, North Haven, CT USA; 22 Division of Physiology, Otto Loewi Research Center of Vascular Biology, Immunity and Inflammation, Medical University of Graz, Graz, Austria; 23 Mohammed Bin Rashid University of Medicine and Applied Health Sciences, Dubai, United Arab Emirates

**Keywords:** Diagnostic markers, Thrombosis, Ultrasonography

Correction to: *npj Microgravity* 10.1038/s41526-024-00356-w, published online 05 February 2024

In this article the main text in Table 6 shall have been the one shown in the PDF file attached. The original article has been corrected.

