# Supplementary material for: Systematic review of the use of ultrasound for venous assessment and venous thrombosis screening in spaceflight
Source: NPJ Microgravity. 2024 Feb 5;10:14. doi: 10.1038/s41526-024-00356-w (PMC10844233; doi:10.1038/s41526-024-00356-w)
Supplement: Supplementary file 1 — SUPPLEMENTARY INFORMATION [file 41526_2024_356_MOESM1_ESM.pdf]

SUPPLEMENTARY INFORMATION

TITLE

Systematic review of the use of ultrasound for venous assessment and venous thrombosis screening in spaceflight.

SUPPLEMENTARY METHODS

Review of studies reporting on upper extremity venous ultrasound on Earth

The review was conducted on diagnostic accuracy and diagnostic management studies for upper extremity deep vein thrombosis (UEDVT) performed on Earth.

The search was performed in PubMed (National Library of Medicine) database using the following combination of terms: ((upper extremity) AND (venous thrombosis) AND (ultrasound) AND (diagnosis) AND ((accuracy) OR (performance))). The search was also carried out using reference lists of retrieved documents and systematic reviews.

Records reporting studies on diagnostic accuracy and diagnostic management of UEDVT in adult patients were eligible for analysis.

The analysis focused on diagnostic performance: (1) in terms of sensitivity, specificity, and area under the receiver operating curves (AUROC) in diagnostic accuracy studies that compared V-US to a reference standard (venography), or (2) in terms of failure rate in diagnostic management studies i.e., the rate of objectively confirmed symptomatic VTE events (i.e., symptomatic deep vein thrombosis (DVT) or pulmonary embolism (PE) or fatal PE) reviewed by an independent clinical adjudication committee, occurring during a 3-month follow-up period in patients with a negative baseline strategy not treated by anticoagulants.

SUPPLEMENTARY TABLES

Supplementary Table 1. Data collected.

Question	Data collected
Who performed and interpreted the US?<br>Image annotation procedures and image capture standards	- Who performed the US during spaceflight (astronaut physician/ sonographer, astronaut with remote control, astronaut with remote guidance, astronaut without remote guidance)? Performing the US includes probe positioning, annotation procedures and image captures.<br>- Astronaut US training information<br>- Who interpreted the US images?
When were measurements performed?<br>(when, how, how long, n of measurements, etc ..)	- Pre-flight,<br>- In-flight (During the flight)<br>- Post-flight
What types of equipment were used?	- Ultrasound equipment device/ scanner<br>- Probes/ transducers used for peripheral veins and central veins<br>o Type (Linear array, Curved array, Phased array, "Baby head"/ Microconvex)<br>o Emission frequency (…, broadband)<br>o Other types
Where was the US performed? (In the upper and lower limbs venous systems)	- Veins and vein segments in neck/ upper limbs venous system<br>o Sides (right side, left side, both sides)<br>o Neck<br>o Upper limb superficial veins<br>o Upper limb deep veins<br>o Intrathoracic veins<br>- Veins and vein segments in lower limbs venous system<br>o Sides (right side, left side, both sides)<br>o Lower limb superficial veins<br>o Lower limb deep veins<br>o Pelvic and abdominal veins<br>- Other venous sites
What ultrasound methods/ modalities were used?	- B mode US and views (transverse, longitudinal)<br>o grayscale images only<br>o grayscale images + compression US<br>- Continuous wave doppler US<br>- Duplex US (B mode + Pulsed wave doppler)<br>- Colour doppler US<br>- Other US methods

**Supplementary Table 1** continued.	
What was assessed/ measured?	- Clinical symptoms and signs<br>- upper extremity, facial and neck<br>- lower extremity<br>- Descriptive B-mode US<br>o Congestion (cephalad venous system dilation),<br>o Increased venous pressures<br>o Stasis/ stagnant flow<br>- Doppler waveform/ flow<br>o Magnitude (e.g. decreased flow in microgravity)<br>o Direction (Forward, Reversed)<br>o Phasicity of spontaneous flow (with cardiac cycle, with respiration)<br>o Augmentation with distal compression<br>o Velocity<br>- Other measurements: Vein diameter - Vein area - Vein pressure - Volume – Ratio - Anything else
How were measurements performed (Standing position, seated position, bedrest, HDBR and degree of HDT, LBNP)?	- Pre-flight<br>- During the flight<br>- Post-flight
What were the venous US study results? (See specific table)	- Venous US study results - Summary of findings
Was a screening for DVT intended to be performed, was a DVT detected and what were the DVT US criteria? (See specific table)	- Was a screening for DVT intended to be performed in the study?<br>- Was a DVT detected?<br>- What were the venous US criteria for the detected DVT?
Were weightlessness countermeasures*used and what types of countermeasures were used, if any? (See specific table)	- Were countermeasures used?<br>- Types of countermeasures, if any?<br>- Effect of countermeasure on venous US findings.
How was the analysis of venous US study results performed?	- Outcome measure<br>- Type of statistical analysis
General and specific comments	- Free general or specific comments about the venous US

NR: Not reported, NA: Not applicable. *Countermeasures (type of countermeasures used: cf Front. Physiol. 2017 Pathophysiology of spaceflight): Flywheel exercise - Vibration exercise - Combined resistive exercise - High-load resistive exercise (RE) - RE+ whole-body vibration - Resistive Vibration Exercise (RVE) - Aerobic exercise - Lower Body Negative Pressure - Reactive jump- Artificial gravity - Neuromuscular electric stimulation - Nutritional supplement - Caloric variations in nutrition - Amino acid infusion - Bisphosphonate – Cocktail - …

**Supplementary Table 2.** Search history - PubMed 2022 07 24		
Search number	Query	Results
1	microgravity	12,413
2	spaceflight	23,398
3	hypogravity	7,583
4	reduced gravity	9,844
5	zero gravity	10,082
6	weightlessness	9,681
7	micro-G	4,008
8	low gravity	11,257
9	zero-G	136
10	astronaut*	10,815
11	cosmonaut*	882
12	#1 OR #2 OR #3 OR #4 OR #5 OR #6 OR #7 OR #8 OR #9 OR #10 OR #11	44,181
13	ultrasound	1,868,780
14	B?mode	37,293
15	echography	1,730,516
16	sonograph*	59,357
17	doppler	147,715
18	#13 OR #14 OR #15 OR #16 OR #17	1,936,878
19	vein	413,609
20	venous	480,803
21	vte	14,056
22	dvt	12,342
23	#19 OR #20 OR #21 OR #22	586,452
24	#12 AND #18 AND #23	165

Supplementary Table 3. Search history - Ovid MEDLINE(R) and Epub Ahead of Print, In-Process, In-Data-Review & Other Non-Indexed Citations, Daily and Versions <1946 to July 26, 2022>

#	Query	Results
1	microgravity.af.	7,150
2	spaceflight.af.	3,834
3	hypogravity.af.	392
4	reduced gravity.af.	306
5	zero gravity.af.	307
6	weightlessness.af.	9,576
7	micro-G.af.	3,942
8	low gravity.af.	201
9	zero-G.af.	132
10	astronaut*.af.	10,875
11	cosmonaut*.af.	883
12	1 or 2 or 3 or 4 or 5 or 6 or 7 or 8 or 9 or 10 or 11	26,256
13	ultrasound.af.	327,109
14	B?mode.af.	78
15	echography.af.	6,459
16	sonograph*.af.	59,460
17	doppler.af.	149,537
18	13 or 14 or 15 or 16 or 17	465,076
19	vein.af.	242,641
20	venous.af.	270,576
21	vte.af.	14,088
22	dvt.af.	12,318
23	19 or 20 or 21 or 22	449,556
24	12 and 18 and 23	66

Supplementary Table 4. Search history – OVID Embase <1974 to 2022 Week 29>

#	Query	Results
1	microgravity.af.	8,443
2	spaceflight.af.	4,072
3	hypogravity.af.	228
4	reduced gravity.af.	322
5	zero gravity.af.	328
6	weightlessness.af.	7,227
7	micro-G.af.	607
8	low gravity.af.	221
9	zero-G.af.	143
10	astronaut*.af.	9,924
11	cosmonaut*.af.	4,021
12	1 or 2 or 3 or 4 or 5 or 6 or 7 or 8 or 9 or 10 or 11	23,899
13	ultrasound.af.	585,169
14	B?mode.af.	280
15	echography.af.	463,863
16	sonograph*.af.	86,115
17	doppler.af.	230,665
18	13 or 14 or 15 or 16 or 17	999,931
19	vein.af.	532,581
20	venous.af.	392,402
21	vte.af.	27,128
22	dvt.af.	23,964
23	19 or 20 or 21 or 22	765,222
24	12 and 18 and 23	96

**Supplementary Table 5.** Search history – OVID EBM Reviews - Cochrane Database of Systematic Reviews <2005 to July 20, 2022>		
**#**	**Query**	**Results**
1	microgravity.af.	0
2	spaceflight.af.	0
3	hypogravity.af.	0
4	reduced gravity.af.	0
5	zero gravity.af.	0
6	weightlessness.af.	2
7	micro-G.af.	10
8	low gravity.af.	1
9	zero-G.af.	0
10	astronaut*.af.	8
11	cosmonaut*.af.	0
12	1 or 2 or 3 or 4 or 5 or 6 or 7 or 8 or 9 or 10 or 11	19
13	ultrasound.af.	1,642
14	B?mode.af.	0
15	echography.af.	66
16	sonograph*.af.	300
17	doppler.af.	482
18	13 or 14 or 15 or 16 or 17	1,856
19	vein.af.	1,056
20	venous.af.	1,358
21	vte.af.	125
22	dvt.af.	166
23	19 or 20 or 21 or 22	1,858
24	12 and 18 and 23	2

**Supplementary Table 6.** Venous ultrasound reported findings in spaceflight.							
Author (Year)	Venous outcome measure	Statistical analysis	US findings	Screening for DVT intended	Was a DVT detected?	DVT US criteria	Spaceflight DVT study comparable to terrestrial DVT study
Arbeille Ph et al (1994)[1]	CSA (LBNP)	NR	Increased jugular (from D1) and femoral (from D9) vein section throughout the duration of the spaceflight, reduced jugular vein distensibility	No	No	NA	NA
Arbeille Ph et al (2001)[2]	CSA	Wilcoxon matched-pairs tests	Increased jugular and portal vein volume, and femoral vein area throughout the duration of the SF as compared to preflight, from week 1 to months 5-6 (as opposed to reduced femoral vein area during HDT)	No	No	NA	NA
Arbeille Ph et al (2015)[3]	CSA	One-way repeated measures analysis of variance with Tukey's post hoc testing (2 excluded from the inflight analysis for missing data)	Increased Jugular and femoral veins, decreased calf veins and gastrocnemius veins	No	No	NA	NA
Arbeille Ph et al (2021)[4]	Morphological & hemodynamic changes (LBNP)	Linear fixed effect models and Linear regression	Increased jugular vein volume and portal vein area, sustained cephalad fluid shift and impact on cerebral venous circulation, LBNP -25 mmHg effective (restored variables at least back to pre-flight supine levels)	No	No	NA	NA

Supplementary Table 6 continued							
Author (Year)	Venous outcome measure	Statistical analysis	US findings	Screening for DVT intended	Was a DVT detected?	DVT US criteria	Spaceflight DVT study comparable to terrestrial DVT study
Auñón-Chancellor, S.M. et al (2020)[5]	Detection of asymptomatic IJV thrombosis and outcome during SF	NA	Asymptomatic left IJV thrombosis during an US examination for a vascular research study. No obvious direct image of the thrombus. Image compatible with a stagnant flow.	No	Yes	Echogenic thrombus (but vein almost compressible and stasis), absence of flow, flow reversal, thining of the image during SF, flow returns to normal on earth	No
David, J. et al (2021)[6]	CSA	Student t test	Right IJV-CSA were significantly different between in-flight values and several angles of the Russian tilt-table protocol, except for the 0° measurement	No	No	NA	NA
Fomina, G.A. et al (2005)[7]	CSA	Mean comparison	Increased jugular vein and femoral vein area throughout the spaceflight, significant difference in femoral area between months 1 and 6, no change in distensibility	No	No	NA	NA
Fomina, G. A. et al (2007)[8]	CSA(Braslets)	Mean comparison	Increased jugular vein and femoral vein area throughout the flight.<br>With "Braslets": Increase of femoral vein area and reduction of cephalic congestion and jugular vein area	No	No	NA	NA
Hamilton, D. R. et al (2012)[9]	CSA (Braslets, Valsalva, Mueller)	Parametric mixed models	Increased jugular and femoral vein area<br>Braslet reduces distention of the jugular venous system and increased sensitivity of the jugular vein area to thoracic maneuvers	No	No	NA	NA
Herault, S. et al (2000)[10]	CSA (LBNP)	Wilcoxon matched-pairs sign-rank test	Without cuffs: Increased jugular and femoral vein area, decreased vascular resistance.<br>With versus without cuffs: decreased jugular area, increased femoral area	No	No	NA	NA

**Supplementary Table 6** continued							
Author (Year)	Venous outcome measure	Statistical analysis	US findings	Screening for DVT intended	Was a DVT detected?	DVT US criteria	Spaceflight DVT study comparable to terrestrial DVT study
Jasien, J. V. et al (2022)[11]	Pressure measurement	Mean difference	D45 inflight pressure not different from pre-flight supine, higher than pre-flight seated, lower than pre-flight 15° HDT, not different from D150	No	No	NA	NA
Lee S. M. C. et al (2020)[12]	CSA, central vein pressure, flow characteristics	Descriptive, linear and logistic regression models, generalized estimating equation	Increased IJV area and pressure with partial gravity acute exposure and description of flow patterns	No	No	NA	NA
Marshall-Goebel, K. et al (2019)[13]	Detection of asymptomatic DVT, IJV CSA, central vein pressure, flow characteristics (LBNP)	Mean comparison	Increased IJV area and pressure with microgravity exposure, description of flow patterns and thrombus formation	No	Yes	Occlusive IJV thrombus (with stagnant flow) and a potential partial IJV thrombus (with stagnant flow) on retrospective analysis	No
Martin, D. S. et al (1996)[14]	IJV pressure	Mean comparison	IJVP was higher in 0G than 1G, increased as gravity levels decreased, was greater in 0G than 1G at all expiration pressures	No	No	NA	NA
Pavela, J. et al (2022)[15]	Detection of asymptomatic DVT, IJV CSA, peak velocity, echogenicity & respiratory manoeuvers	Evaluation by matching terrestrial and in-flight ultrasounds.	No DVT detected, decreased peak velocity in both IJV, increased CSA, increased blood echogenicity most frequently in the left IJV, and slow, retrograde left IJV blood flow in two astronauts	Yes	No	No DVT detected	No

Supplementary Table 6 continued							
Author (Year)	Venous outcome measure	Statistical analysis	US findings	Screening for DVT intended	Was a DVT detected?	DVT US criteria	Spaceflight DVT study comparable to terrestrial DVT study
Zamboni, P. et al (2018)[16]	Diagnostic accuracy and values of IJV pulse trace parameters	Sensitivity, specificity and accuracy - mean and standard deviations	Diagnostic accuracy did not significantly differ from that on earth, parameters values increased by 15% in the first ISS session but decreased by 50% in the second ISS session.<br>Many study limitations in the design, conduct, analysis and reporting.	No	No	NA	NA

Between parentheses, the additional countermeasures used. *LBNP*: lower body negative pressure, *DVT*: deep venous thrombosis, *IJV*: internal jugular vein, *CSA*: cross sectional area

**Supplementary Table 7.** Effect of in-flight venous interventions.			
Author (Year)	Interventions	Type of intervention	Effect of interventions
Arbeille Ph et al (1994)[1]	Yes	LBNP (-25 mmHg and -45mmHg of 10 min with a transition step of -35 mmHg)	No assessment of the IJV response to LBNP (n= 1).<br>The cerebral flow and resistance decreased moderately (use of inflatable cuffs/ bracelets reported). The calf volume increased as the LBNP pressure decreased.
Arbeille Ph et al (2001)[2]	NR	NA	NA
Arbeille Ph et al (2015)[3]	No	NA	NA
Arbeille Ph et al (2021)[4]	Yes	LBNP (-25 mmHg) for 30 minutes	Quantitative data analyses using continuous variables (n=14).<br>LBNP restored variables at least back to pre-flight supine levels.<br>From figure: IJV volume returns to pre-flight supine level in 10/ 12 astronauts.
Auñón-Chancellor, S.M. et al (2020)[5]	Yes	Mueller manoeuvre	Flow through the affected IJV induced by Mueller manoeuvre (n= 1 astronaut).
David, J. et al (2021)[6]	No	NA	NA
Fomina, G.A. et al (2005)[7]	No	NA	NA
Fomina, G. A. et al (2007)[8]	Yes	Bracelets	Quantitative data analysis (continuous variables) (n= 6).<br>Reduction of cephalic congestion and jugular vein area.<br>No noticeable effect on cerebral blood flow.<br>Femoral vein area higher
Hamilton, D. R. et al (2012)[9]	Yes	Bracelets +/- Valsalva +/- Mueller	Quantitative data analysis (continuous variables) (n= 9).<br>Increased femoral vein area with bracelets, little additional increase with bracelets + Valsalva, increased femoral vein with Valsalva, decreased IJV area with bracelets, Mueller and both bracelets + Mueller
Herault, S. et al (2000)[10]	Yes	LBNP (-25 mmHg, -35 mmHg and -45mmHg)	Quantitative data analysis (continuous variables) (n= 6).<br>With versus without cuffs (bracelets): decreased jugular area, increased femoral area.<br>LBNP -45 mmHg: Less increase of calf circumference, femoral resistance, and cerebral/femoral blood flow ratio inflight and postflight than pre-flight.
Jasien, J. V. et al (2022)[11]	Yes	LBNP (-25 mmHg)	Quantitative data analysis (continuous variables) (n= 13)<br>No assessment of the IJV response to LBNP.
Lee S. M. C. et al (2020)[12]	No	NA	NA

**Supplementary Table 7** continued			
Author (Year)	Interventions	Type of intervention	Effect of interventions
Marshall-Goebel, K. et al (2019)[13]	Yes	LBNP (-25mmHg)	Quantitative data analysis (continuous variables) (n= 11)<br>Stagnant or reverse flow in the IJV was observed in 6/ 11 crewmembers (55%)<br>LBNP -25 mmHg during spaceflight reduced IJV area and improved blood flow in 10 of 17 sessions (59%)
Martin, D. S. et al (1996)[14]	Yes	Respiratory manoeuvres (controlled Valsalva manoeuvres)	Quantitative data analysis (continuous variables) (n= 11)<br>IJV pressure measurements performed under respiratory manoeuvres in 7/ 11 crewmembers. IJV pressure appears to increase as the level of gravity decreases.
Pavela, J. et al (2022)[15]	Yes	Respiratory maneuvers (Valsalva, modified Mueller, contralateral IJV compression)	Quantitative data analysis (continuous variables) (n= 11)<br>Modified Mueller increased peak velocity and reduced blood flow echogencity<br>In two individuals with retrograde blood flow, both the modified Mueller manoeuvre and the contralateral manual compression of the right IJV reversed the flow direction to antegrade while the manoeuvre was performed.
Zamboni, P. et al (2018)[16]	No	NA	NA

**Supplementary Table 8.** Crew performing the venous ultrasound assessment.									
Author (Year)	Staff who carried out the SF US	Astronaut US training (and training type)	On-earth remote manual control	Comments on staff performing the US	Image annotation procedure	Image capture standards	Who interpreted the US	Issues/ limitations in performing SF US	Comparability to terrestrial staff performing the US
Arbeille Ph et al (1994)[1]	NR	NR	NR	Reporting issues	NR	NR	Sonographer	Reporting issues	Not comparable
Arbeille Ph et al (2001)[2]	NR	NR	NR	Reporting issues	NR	NR	Sonographer	Reporting issues	Not comparable
Arbeille Ph et al (2015)[3]	Astronaut	Yes (3h for inflight image capture)	No	Remote guidance	Remote guidance	Yes	Sonographer	Remote guidance	Not comparable
Arbeille Ph et al (2021)[4]	Astronaut	Yes (3 hours for inflight measures)	No	Remote guidance	Remote guidance	Yes	Sonographer	Remote guidance	Not comparable
Aunon S.M. et al (2020)[5]	Physician astronaut	NA	No	Extent of training for DVT screening unknown	Physician astronaut	Yes	Sonographer	Unknown expertise in DVT screening	Not comparable
David, J. et al (2021)[6]	Astronaut	NR	NR	Reporting issues	NR	NR	Sonographer	Reporting issues	Not comparable
Fomina, G.A. et al (2005)[7]	Physician astronaut	NR	NR	Expert	Sonographer	Yes	NR	Reporting issues on interpretation of US but US performed by sonographer	Comparable
Fomina, G. A. et al (2007)[8]	Physician astronaut	NR	NR	Expert	Sonographer	Yes	NR	Reporting issues on interpretation of US but US performed by sonographer	Comparable
Hamilton et al (2012)[9]	Astronaut	Yes (Familiarization)	No	Remote guidance	Remote guidance	Yes	Sonographer	Remote guidance	Not comparable

Supplementary Table 8 continued									
Author (Year)	Staff who carried out the SF US	Astronaut US training (and training type)	On-earth remote manual control	Comments on staff performing the US	Image annotation procedure	Image capture standards	Who interpreted the US	Issues/ limitations in performing SF US	Comparability to terrestrial staff performing the US
Herault, S. et al (2000)[10]	NR	NR	NR	Reporting issues	NR	NR	Sonographer	Reporting issues	Not comparable
Jasien, J. V. et al (2022)[11]	NR	NR	NR	Reporting issues	NR	NR	Sonographer	Reporting issues (pressure measurement using US)	Not comparable
Lee S. M. C. et al (2020)[12]	Sonographer	NA	No	Expert	Sonographer	Yes	Two to three sonographers	No limitations	Comparable
Marshall-Goebel, K. et al (2019)[13]	Physician astronaut	NA	No	Extent of training for DVT screening unknown	Physician astronaut	Yes	Two to three sonographers	Unknown expertise in DVT screening	Not comparable
Martin, D. S. et al (1996)[14]	Sonographer	NA	No	Expert	Sonographer	Yes	Sonographer	No limitations	Comparable
Pavela, J. et al (2022)[15]	Astronaut	NR (but likely)	No	Remote guidance	Remote guidance	Yes	Vascular team	Remote guidance	Not comparable
Zamboni, P. et al (2018)[16]	Astronaut	Yes	NR	Reporting issues	NR	Yes	Sonographer	Reporting issues	Not comparable

SF: spaceflight, *US*: ultrasound, *NR*: Not reported, *NA*: Not applicable, *Remote guidance*: verbal/ voice remote control

Staff performing SF US (see definitions in text):

- Expert physician astronaut or sonographer on board (n= 6, two of which reporting on same IJV thrombosis case but expertise of astronaut for V-US upper extremity DVT screening is unknown), Remote control (manually, n= 0), Remote guidance (verbal, n= 4), Reporting issues (n= 6)
- Issues/ limitations in performing SF US: No limitations (n= 2), Reporting issues (n= 8, two of which only on personnel interpreting the US but US performed by sonographer), Remote guidance (n= 6)

SF staff performing the US comparable to terrestrial one i.e., US performed and interpreted by physician/ sonographer on board expert in the field of V-US DVT studies or other studies (n=4), or via remote (manual) control (n=0).

**Supplementary Table 9.** Equipment used for venous ultrasound assessment.						
Author (Year)	Device	Peripheral veins – US emission frequency	Central veins - US emission frequency	US modality	Equipment issues/ limitations	Explanations for non-comparability of SF US equipment versus terrestrial one
Arbeille Ph et al (1994)[1]	"As de cœur"	5 Mhz	3.5 Mhz	CW + Duplex	Reporting issues (published at a conference meeting)	Old study published in 1994
Arbeille Ph et al (2001)[2]	NR	NR	S3 Mhz	B-mode + Doppler	Summary of multiple records	Summary of multiple studies published in 1988, 1992, 1994, 1996, 1997, 1998, 1999 and 2000
Arbeille Ph et al (2015)[3]	NR	NR	NR	B-mode	Only B-mode US used (and Reporting issues)	Only B-mode US used
Arbeille Ph et al (2021)[4]	Vivid Q, GE	NR	NA	B-mode + CDUS + Duplex	Reporting issues	Reporting issues
Aunon S.M. et al (2020)[5]*	Vivid Q, GE	L12 Mhz	NA	B-mode + CDUS + Duplex	No limitation	For DVT detection: Need for lower US emission frequency and smaller US probes to examine deeper vein segments (otherwise no limitation)
David, J. et al (2021)[6]	Butterfly	Linear array (Mhz NR)	NA	B-mode	Only B-mode US used	Only B-mode US used
Fomina, G.A. et al (2005)[7]	Echograph 2M	NR	NA	B-mode + Duplex	Reporting issues	Old studies and reporting issues (Summary and analysis)
Fomina, G. A. et al (2007)[8]	Echograph 2M	NR	NA	B-mode + Duplex	Reporting issues	Old study and reporting issues
Hamilton et al (2012)[9]	ATL HDI-5000	L12-5 Mhz	NA	B-mode	Only B-mode US used	Only B-mode US used
Herault, S. et al (2000)[10]	Matra	C5 Mhz	S3 Mhz	B-mode + CW doppler	Old study	Old study, old equipment (5 Mhz probe used for IJV and femoral vein studies)
Jasien, J. V. et al (2022)[11]	Vivid Q, GE and VeinPress	L12-5 Mhz	NA	B-mode + other modality	Pressure measurements using different equipment	Different equipment for a different purpose
Lee S. M. C. et al (2020)[12]	Vivid Q, GE	L12-5 Mhz	P4	B-mode + CUS + Duplex	No limitation	Different equipment for a different purpose
Marshall-Goebel, K. et al (2019)[13]**	Vivid Q, GE	L12-5 Mhz	NA	B-mode + CUS + Duplex	No limitation	For DVT detection: Need for lower US emission frequency and smaller US probes to examine deeper vein segments (otherwise no limitation)

**Supplementary Table 9** continued						
Author (Year)	Device	Peripheral veins – US emission frequency	Central veins - US emission frequency	US modality	Equipment issues/ limitations	Explanations for non-comparability of SF US equipment versus terrestrial one
Martin, D. S. et al (1996)[14]	Vivid Q, GE	L12-5 Mhz	NA	B-mode + CUS	No limitation	Different equipment for different purpose
Pavela, J. et al (2022)[15]**	Vivid Q, GE	L12-5 Mhz	NA	B-mode + Duplex	No limitation	For DVT detection: Need for lower US emission frequency and smaller US probes to examine deeper vein segments (otherwise no limitation)
Zamboni, P. et al (2018)[16]	Vivid Q, GE	NR	NA	B-mode	Reporting issues	Different purpose (assessment of IJV pulse)

*Whole publication related to DVT detection, ** Part of publication related to DVT detection. *SF*: spaceflight, *US*: ultrasound, *NR*: Not reported, *NA*: Not applicable, *CW*: Continuous wave doppler US, *Duplex*: B-mode with pulsed wave doppler US, *CDUS*: Colour doppler ultrasound. For studies involved with DVT detection, the emission frequency is too high and the probe too large (linear) to enable to investigate deeper vein segments (deeper internal jugular and subclavian veins, intrathoracic veins).

Supplementary Table 10. Venous ultrasound assessment sites.								
Author (Year)	Neck/ upper limb/ intra-thoracic veins			Lower limb/ abdomen/ pelvis veins			Issues/ limitations in venous sites assessment	Explanations for non-comparability of SF US versus terrestrial venous sites assessment
	Vein segments assessed	Vein assessment side	Views used for venous assessment	Vein segments assessed	Site of venous assessment	Views used for venous assessment		
Arbeille Ph et al (1994)[1]	IJV	NR	NR	Femoral vein	NR	NR	Yes	Assessment limited to IJV and femoral veins
Arbeille Ph et al (2001)[2]	IJV	NR	Transverse	Femoral vein	NR	Transverse	Yes	Assessment limited to IJV and femoral veins
Arbeille Ph et al (2015)[3]	IJV	NR	Both	Femoral vein, gastrocnemius vein, tibial vein	NR	NR	Yes	Assessment limited to IJV and femoral veins (and other LL vein segments)
Arbeille Ph et al (2021)[4]	IJV	Left	Both	NA	NA	NA	Yes	Assessment limited to IJV vein (and middle cerebral vein)
Aunon S.M. et al (2020)[5]	IJV, subclavian and axillary veins (2 sides)	Both	NR	Popliteal and femoral veins (2 sides)	Both	NA	Yes	No assessment of intrathoracic veins
David, J. et al (2021)[6]	IJV	Right	Transverse	NA	NA	NA	Yes	Assessment limited to IJV
Fomina, G.A. et al (2005)[7]	IJV	NR	NR	Femoral, hepatic, portal and spleen veins	NR	NR	Yes	Assessment limited to IJV and femoral veins
Fomina, G. A. et al (2007)[8]	IJV	NR	NR	Femoral vein	NR	NR	Yes	Assessment limited to IJV and femoral veins
Hamilton et al (2012)[9]	IJV	NR	Transverse	Femoral vein	NR	NR	Yes	Assessment limited to IJV and femoral veins
Herault, S. et al (2000)[10]	IJV	NR	Transverse	Femoral vein	NR	Transverse	Yes	Assessment limited to IJV and femoral veins
Jasien, J. V. et al (2022)[11]	IJV	NR	NR	NA	NA	NA	YES	Assessment limited to IJV for a different purpose

**Supplementary Table 10** continued								
Author (Year)	Neck/ upper limb/ intra-thoracic veins			Lower limb/ abdomen/ pelvis veins			Issues/ limitations in venous sites assessment	Explanations for non-comparability of SF US versus terrestrial venous sites assessment
	Vein segments assessed	Vein assessment side	Views used for venous assessment	Vein segments assessed	Site of venous assessment	Views used for venous assessment		
Lee S. M. C. et al (2020)[12]	IJV	Right	NR	NA	NA	NA	Yes	Assessment limited to IJV for a different purpose
Marshall-Goebel, K. et al (2019)[13]	IJV	Left	NR	NA	NA	NA	Yes	Assessment limited to IJV
Martin, D. S. et al (1996)[14]	IJV	Right	NR	NA	NA	NA	Yes	Assessment limited to IJV for a different purpose
Pavela, J. et al (2022)[15]	IJV	Both	NR	NA	NA	NA	Yes	Assessment limited to IJV
Zamboni, P. et al (2018)[16]	IJV	Both	NR	NA	NA	NA	Yes	Assessment limited to IJV for a different purpose

*Whole publication related to DVT detection, ** Part of publication related to DVT detection. *IJV*: Internal jugular vein, *SF*: spaceflight, *US*: ultrasound, *NR*: Not reported, *NA*: Not applicable. In the upper limb venous network, assessment was limited to the internal jugular vein mostly for different purposes. In DVT studies, intrathoracic veins were not examined.

**Supplementary Table 11.** Venous ultrasound assessment methods/ measurements							
Author (Year)	Neck/ Upper limb venous US				Lower limb venous US		Issues/ limitations that explain no comparability to on-earth venous US assessment
	S & S / B-mode US cephalad congestion	Doppler US	Vein measurement	Anything else	S & S	Changes	
Arbeille Ph et al (1994)[1]	S & S: NR<br>Congestion: Yes<br>Increased VP: Yes<br>Stasis: NR	Magnitude: Decreased<br>Flow direction: NR<br>Phasicity: NR<br>Distal compression: NA	Diameter: NR<br>CSA: Yes<br>Volume: NR<br>Distensibility: Yes	Vein return index (S/D) reduced	NR	Femoral v. CSA enlarged	Descriptive findings of morphology and velocity changes limited to IJV and femoral veins
Arbeille Ph et al (2001)[2]	S & S: Yes<br>Congestion: Yes<br>Increased VP: NR<br>Stasis: NR	Magnitude: NR<br>Flow direction: NR<br>Phasicity: NR<br>Distal compression: NR	Diameter: NR<br>CSA: Yes<br>Volume: NR<br>Distensibility: NR	No	NR	Femoral v. CSA enlarged	Descriptive findings limited to IJV and femoral vein area measurements
Arbeille Ph et al (2015)[3]	S & S: NR<br>Congestion: Yes<br>Increased VP: NA<br>Stasis: NR	Magnitude: NA<br>Flow direction: NA<br>Phasicity: NA<br>Distal compression: NA	Diameter: NR<br>CSA: Yes<br>Volume: Yes<br>Distensibility: NR	Portal vein studies	NR	Increase of femoral v. & decrease of calf v. & gastrocnemius v.	Descriptive findings of morphology limited to IJV and femoral (and other LL) veins
Arbeille Ph et al (2021)[4]	S & S: NR<br>Congestion: Yes<br>Increased VP: NA<br>Stasis: NR	Magnitude: NR<br>Flow direction: NR<br>Phasicity: NR<br>Distal compression: NA	Diameter: NR<br>CSA: Yes<br>Volume: Yes<br>Distensibility: NR	Portal vein and middle cerebral vein studies	NA	NA	Descriptive findings of morphology and velocity changes limited to IJV
Aunon S.M. et al (2020)[5]	S & S: Yes<br>Congestion: Yes<br>Increased VP: NA<br>Stasis: Yes	Magnitude: NR<br>Flow direction: Reversed<br>Phasicity: Abnormal*<br>Distal compression: Yes	Diameter: NR<br>CSA: NR<br>Volume: NR<br>Distensibility: NA	Image of thrombus (but image not so obvious)	NA	NA	Intrathoracic veins not examined, IJV thrombus image provided not so obvious (comments in text)
David, J. et al (2021)[6]	S & S: NR<br>Congestion: Yes<br>Increased VP: NR<br>Stasis: NR	Magnitude: NR<br>Flow direction: NR<br>Phasicity: NR<br>Distal compression: NR	Diameter: NR<br>CSA: Yes<br>Volume: NR<br>Distensibility: NR	No	NA	NA	Descriptive findings limited to IJV and femoral vein area measurements
Fomina, G.A. et al (2005)[7]	S & S: Yes<br>Congestion: Yes<br>Increased VP: NR<br>Stasis: Yes	Magnitude: NR<br>Flow direction: NR<br>Phasicity: NR<br>Distal compression: NR	Diameter: NR<br>CSA: Yes<br>Volume: NR<br>Distensibility= No	No	NR	Femoral v. CSA enlarged	Descriptive findings limited to IJV and femoral vein area measurements

**Supplementary Table 11** continued							
Author (Year)	Neck/ Upper limb venous US				Lower limb venous US		Issues/ limitations that explain no comparability to on-earth venous US assessment
	S & S / B-mode US cephalad congestion	Doppler US	Vein measurement	Anything else	S & S	Changes	
Fomina, G. A. et al (2007)[8]	S & S: Yes<br>Congestion: Yes<br>Increased VP: NR<br>Stasis: Yes	Magnitude: NR<br>Flow direction: NR<br>Phasicity: NR<br>Distal compression: NR	Diameter: NR<br>CSA: Yes<br>Volume: NR<br>Distensibility: NR	No	NR	Femoral v. CSA enlarged	Descriptive findings limited to IJV and femoral vein area measurements
Hamilton et al (2012)[9]	S & S: NR<br>Congestion: Yes<br>Increased VP: NR<br>Stasis: NR	Magnitude: NR<br>Flow direction: NR<br>Phasicity: NR<br>Distal compression: NR	Diameter: Yes<br>CSA: Yes<br>Volume: NR<br>Distensibility: NR	No	NR	Femoral v. CSA enlarged	Descriptive findings limited to IJV and femoral vein area measurements
Herault, S. et al (2000)[10]	S & S: Yes<br>Congestion: Yes<br>Increased VP: NR<br>Stasis: NR	Magnitude: NR<br>Flow direction: NR<br>Phasicity: NR<br>Distal compression: NR	Diameter: NR<br>CSA: Yes<br>Volume: NR<br>Distensibility: NR	No	NR	Femoral v. CSA enlarged	Descriptive findings limited to IJV and femoral vein area measurements
Jasien, J. V. et al (2022)[11]	S & S: NR<br>Congestion: NR<br>Increased VP: Yes<br>Stasis: NR	Magnitude: NR<br>Flow direction: NR<br>Phasicity: NR<br>Distal compression: NR	Diameter: NR<br>CSA: NR<br>Volume: NR<br>Distensibility: NR	Vein pressure measurement	NR	NA	Different study objective
Lee S. M. C. et al (2020)[12]	S & S: NA<br>Congestion: No<br>Increased VP: Yes<br>Stasis: Yes	Magnitude: NR<br>Flow direction: Flow patterns<br>Phasicity: Normal*<br>Distal compression: NR	Diameter: NR<br>CSA: Yes<br>Volume: NR<br>Distensibility: NR	Vein pressure measurement	NA	NA	Different study objective
Marshall-Goebel, K. et al (2019)[13]	S & S: NA<br>Congestion: Yes<br>Increased VP: Yes<br>Stasis: Yes	Magnitude: Decreased<br>Flow direction: Flow patterns<br>Phasicity: Normal*<br>Distal compression: NA	Diameter: NR<br>CSA: Yes<br>Volume: NR<br>Distensibility: NR	Vein pressure measurement	NA	NA	Intrathoracic veins not examined
Martin, D. S. et al (1996)[14]	S & S: NA<br>Congestion: NR<br>Increased VP: Yes<br>Stasis: NR	Magnitude: NR<br>Flow direction: NR<br>Phasicity: NR<br>Distal compression: NR	Diameter: NR<br>CSA: NR<br>Volume: NR<br>Distensibility: NR	Vein pressure measurement	NA	NA	Different study objective

**Supplementary Table 11** continued							
Author (Year)	Neck/ Upper limb venous US				Lower limb venous US		Issues/ limitations that explain no comparability to on-earth venous US assessment
	S & S / B-mode US cephalad congestion	Doppler US	Vein measurement	Anything else	S & S	Changes	
Pavela, J. et al (2022)[15]	S & S: NA<br>Congestion: Yes<br>Increased VP: NR<br>Stasis: Yes	Magnitude: Decreased<br>Flow direction: Reversed<br>Phasicity: NR<br>Distal compression: NR	Diameter: NR<br>CSA: Yes<br>Volume: NR<br>Distensibility: NR	Spontaneous echo contrast echogenicity	NA	NA	Intrathoracic veins not examined
Zamboni, P. et al (2018)[16]	S & S: NR<br>Congestion: NR<br>Increased VP: NR<br>Stasis: NR	Magnitude: NR<br>Flow direction: NR<br>Phasicity: NR<br>Distal compression: NR	Diameter: NR<br>CSA: NR<br>Volume: NR<br>Distensibility: NR	Derivation of IJV pulse trace from B-mode US	NA	NA	Different study objective

S & S: symptoms and signs, Congestion: dilation, *VP*: venous pressure, *Stasis*: slow flow on B-mode, *Phasicity*: phasicity of spontaneous flow, *Distal compression*: augmentation with distal compression, *v.*: vein-s-, *CSA*: cross sectional area, *Phasicity*: Normal (with cardiac/ respiratory cycles) - Abnormal (loss of phasicity).

Assessment methods

Cephalad congestion was based either on symptoms and signs or on B-mode US (dilation, increased vein pressure or stasis).

Doppler US was rarely assessed (n=4). The various reported flow patterns (decreased magnitude, reversed flow direction, different flow aspects, abnormal phasicity) were not examined or reported systematically in studies

Reported findings

- *Cephalad congestion symptoms and signs* (n= 5)
- *Cephalad congestion B-mode US*:
  - - Dilation (n= 12) - Increased venous pressure (n= 5) - Stasis pattern (spontaneous echo contrast) (n= 6) -At least a single type of congestion (n= 11)
- *Doppler US*:
  - - Magnitude: Decreased (n= 3) – Flow: Reversed (n= 2), Different flow patterns described (n= 2) - Phasicity of spontaneous flow: Assessed (n= 3), loss of phasicity (n= 1 out of 3) - Augmentation with distal compression (n=1)
- *Vein measurements*:
  - - Vein diameter: Increased (n= 1) - Vein CSA: Increased (n= 12) - Vein volume: Increased (n= 2) - Vein distensibility: Decreased (n= 1), not decreased (n=1)
- *Anything else*: Reduced vein return index: n= 1 - Portal vein (n=1) - Portal and middle cerebral vein studies - Derivation of IJV pulse trace from a B-mode US assessment

SUPPLEMENTARY FIGURE

PRISMA 2020 Checklist

Section and Topic	Item #	Checklist item	Location where item is reported
**TITLE**			
Title	1	Identify the report as a systematic review.	Title
**ABSTRACT**			
Abstract	2	See the PRISMA 2020 for Abstracts checklist.	Text 5
**INTRODUCTION**			
Rationale	3	Describe the rationale for the review in the context of existing knowledge.	Text 6,7
Objectives	4	Provide an explicit statement of the objective(s) or question(s) the review addresses.	Text 8
**METHODS**			
Eligibility criteria	5	Specify the inclusion and exclusion criteria for the review and how studies were grouped for the syntheses.	Text 8<br>Table 1
Information sources	6	Specify all databases, registers, websites, organisations, reference lists and other sources searched or consulted to identify studies. Specify the date when each source was last searched or consulted.	Text 8
Search strategy	7	Present the full search strategies for all databases, registers and websites, including any filters and limits used.	Text 9<br>Table 2
Selection process	8	Specify the methods used to decide whether a study met the inclusion criteria of the review, including how many reviewers screened each record and each report retrieved, whether they worked independently, and if applicable, details of automation tools used in the process.	Text 9
Data collection process	9	Specify the methods used to collect data from reports, including how many reviewers collected data from each report, whether they worked independently, any processes for obtaining or confirming data from study investigators, and if applicable, details of automation tools used in the process.	Text 9,10
Data items	10a	List and define all outcomes for which data were sought. Specify whether all results that were compatible with each outcome domain in each study were sought (e.g. for all measures, time points, analyses), and if not, the methods used to decide which results to collect.	Text 9,10<br>Table S1
	10b	List and define all other variables for which data were sought (e.g. participant and intervention characteristics, funding sources). Describe any assumptions made about any missing or unclear information.	Text 9,10<br>Table S1
Study risk of bias assessment	11	Specify the methods used to assess risk of bias in the included studies, including details of the tool(s) used, how many reviewers assessed each study and whether they worked independently, and if applicable, details of automation tools used in the process.	Text 10, 12, 22,23

Section and Topic	Item #	Checklist item	Location where item is reported
Effect measures	12	Specify for each outcome the effect measure(s) (e.g. risk ratio, mean difference) used in the synthesis or presentation of results.	Text 10, 11 (NA)
Synthesis methods	13a	Describe the processes used to decide which studies were eligible for each synthesis (e.g. tabulating the study intervention characteristics and comparing against the planned groups for each synthesis (item #5)).	Text 10, 11 (NA)
	13b	Describe any methods required to prepare the data for presentation or synthesis, such as handling of missing summary statistics, or data conversions.	Text 10, 11 (NA)
	13c	Describe any methods used to tabulate or visually display results of individual studies and syntheses.	Text 10, 11 (NA)
	13d	Describe any methods used to synthesize results and provide a rationale for the choice(s). If meta-analysis was performed, describe the model(s), method(s) to identify the presence and extent of statistical heterogeneity, and software package(s) used.	Text 10, 11 (NA)
	13e	Describe any methods used to explore possible causes of heterogeneity among study results (e.g. subgroup analysis, meta-regression).	Text 10, 11 (NA)
	13f	Describe any sensitivity analyses conducted to assess robustness of the synthesized results.	Text 10, 11 (NA)
Reporting bias assessment	14	Describe any methods used to assess risk of bias due to missing results in a synthesis (arising from reporting biases).	Text 12
Certainty assessment	15	Describe any methods used to assess certainty (or confidence) in the body of evidence for an outcome.	Text 10, 11 (NA)
**RESULTS**			
Study selection	16a	Describe the results of the search and selection process, from the number of records identified in the search to the number of studies included in the review, ideally using a flow diagram.	Text 11 Tables S2-S5 Figure 1
	16b	Cite studies that might appear to meet the inclusion criteria, but which were excluded, and explain why they were excluded.	Text 11 Figure 1
Study characteristics	17	Cite each included study and present its characteristics.	Text 11, 12 Table 3
Risk of bias in studies	18	Present assessments of risk of bias for each included study.	Text 12, 22, 23
Results of individual studies	19	For all outcomes, present, for each study: (a) summary statistics for each group (where appropriate) and (b) an effect estimate and its precision (e.g. confidence/credible interval), ideally using structured tables or plots.	(NA)
Results of	20a	For each synthesis, briefly summarise the characteristics and risk of bias among contributing studies.	Text 12, 22,

Section and Topic	Item #	Checklist item	Location where item is reported
syntheses			23<br>Text 14 – 21<br>Tables 4 – 7<br>Tables S6-S11<br>(NA)
	20b	Present results of all statistical syntheses conducted. If meta-analysis was done, present for each the summary estimate and its precision (e.g. confidence/credible interval) and measures of statistical heterogeneity. If comparing groups, describe the direction of the effect.	Text 14 – 21<br>Tables 4 – 7<br>Tables S6-S11<br>(NA)
	20c	Present results of all investigations of possible causes of heterogeneity among study results.	(NA)
	20d	Present results of all sensitivity analyses conducted to assess the robustness of the synthesized results.	(NA)
Reporting biases	21	Present assessments of risk of bias due to missing results (arising from reporting biases) for each synthesis assessed.	(NA)
Certainty of evidence	22	Present assessments of certainty (or confidence) in the body of evidence for each outcome assessed.	(NA)
**DISCUSSION**			
Discussion	23a	Provide a general interpretation of the results in the context of other evidence.	Text 21, 22
	23b	Discuss any limitations of the evidence included in the review.	Text 22, 23
	23c	Discuss any limitations of the review processes used.	Text 23
	23d	Discuss implications of the results for practice, policy, and future research.	Text 24-26
**OTHER INFORMATION**			
Registration and protocol	24a	Provide registration information for the review, including register name and registration number, or state that the review was not registered.	Abstract<br>Text 7
	24b	Indicate where the review protocol can be accessed, or state that a protocol was not prepared.	Text 7
	24c	Describe and explain any amendments to information provided at registration or in the protocol.	None
Support	25	Describe sources of financial or non-financial support for the review, and the role of the funders or sponsors in the review.	Text 28
Competing interests	26	Declare any competing interests of review authors.	Text 28

Section and Topic	Item #	Checklist item	Location where item is reported
Availability of data, code and other materials	27	Report which of the following are publicly available and where they can be found: template data collection forms; data extracted from included studies; data used for all analyses; analytic code; any other materials used in the review.	Text 28

NA: Not applicable

From: Page MJ, McKenzie JE, Bossuyt PM, Boutron I, Hoffmann TC, Mulrow CD, et al. The PRISMA 2020 statement: an updated guideline for reporting systematic reviews. BMJ 2021;372:n71. doi: 10.1136/bmj.n71

For more information, visit: http://www.prisma-statement.org/

SUPPLEMENTARY REFERENCES

1. Arbeille, P., Pottier, J.M., Fomina, G., Roncin, A. & Kotovskaya, A. Cardiac & vascular response to 0.g exposure during the 14-day "Antares" Sapceflight. *Proceedings 5th Eur. Symp. on "Life Sciences Research in Space", Arcachon, France, 26 Sept. - 1st Oct. 1993 (ESA SP-366, August 1994)* (1994).
2. Arbeille, P.*, et al.* Adaptation of the left heart, cerebral and femoral arteries, and jugular and femoral veins during short- and long-term head-down tilt and spaceflights. *Eur J Appl Physiol* **86**, 157-168 (2001).
3. Arbeille, P., Provost, R., Zuj, K. & Vincent, N. Measurements of jugular, portal, femoral, and calf vein cross-sectional area for the assessment of venous blood redistribution with long duration spaceflight (Vessel Imaging Experiment). *Eur J Appl Physiol* **115**, 2099-2106 (2015).
4. Arbeille, P.*, et al.* Lower body negative pressure reduces jugular and portal vein volumes and counteracts the elevation of middle cerebral vein velocity during long-duration spaceflight. *J Appl Physiol (1985)* **131**, 1080-1087 (2021).
5. Aunon, S.M., Pattarini, J.M., Moll, S. & Sargsyan, A. Venous Thrombosis during Spaceflight. *New England Journal of Medicine* **382(1)**, 89-90 (2020).
6. David, J.*, et al.* Comparison of Internal Jugular Vein Cross-Section Area During a Russian Tilt-Table Protocol and Microgravity. *Aerosp Med Hum Perform* **92**, 207-211 (2021).
7. Fomina, G.A. & Kotovskaia, A.R. [Shifts in human venous hemodynamics in long-term space flight]. *Aviakosm Ekolog Med* **39**, 25-30 (2005).
8. Fomina, G.A., Kotovskaia, A.R. & Temnova, E.V. [Effect of prophylactic device "Braslet" on hemodynamic changes during long-term missions to orbital station Mir]. *Aviakosm Ekolog Med* **41**, 8-13 (2007).
9. Hamilton, D.R.*, et al.* Cardiac and vascular responses to thigh cuffs and respiratory maneuvers on crewmembers of the International Space Station. *J Appl Physiol (1985)* **112**, 454-462 (2012).
10. Herault, S.*, et al.* Cardiac, arterial and venous adaptation to weightlessness during 6-month MIR spaceflights with and without thigh cuffs (bracelets). *Eur J Appl Physiol* **81**, 384-390 (2000).
11. Jasien, J.V.*, et al.* Noninvasive Indicators of Intracranial Pressure Before, During, and After Long-Duration Spaceflight. *J Appl Physiol (1985)* (2022).
12. Lee, S.M.C.*, et al.* Venous and Arterial Responses to Partial Gravity. *Front Physiol* **11**, 863 (2020).
13. Marshall-Goebel, K.*, et al.* Assessment of Jugular Venous Blood Flow Stasis and Thrombosis During Spaceflight. *JAMA netw* **2**, e1915011 (2019).
14. Martin, D.S.*, et al.* Internal jugular pressure increases during parabolic flight. *Physiological Reports* **4(24) (no pagination)**(2016).
15. Pavela, J.*, et al.* Surveillance for jugular venous thrombosis in astronauts. *Vasc Med*, 1358863x221086619 (2022).
16. Zamboni, P.*, et al.* Ultrasound Monitoring of Jugular Venous Pulse during Space Missions: Proof of Concept. *Ultrasound Med Biol* **44**, 726-733 (2018).
