# Supplementary material for: Systematic review of the use of ultrasound for venous assessment and venous thrombosis screening in spaceflight
Source: NPJ Microgravity. 2024 Feb 5;10:14. doi: 10.1038/s41526-024-00356-w (PMC10844233; doi:10.1038/s41526-024-00356-w)
Supplement: Supplementary file 2 — Reporting Summary [file 41526_2024_356_MOESM2_ESM.pdf]

nature portfolio

Corresponding author(s): DAPR NPJMGRAV-01119R

Last updated by author(s): Nov 29, 2023

Reporting Summary

Nature Portfolio wishes to improve the reproducibility of the work that we publish. This form provides structure for consistency and transparency in reporting. For further information on Nature Portfolio policies, see our Editorial Policies and the Editorial Policy Checklist.

Statistics

For all statistical analyses, confirm that the following items are present in the figure legend, table legend, main text, or Methods section.

n/a	Confirmed	
☒	☐	The exact sample size (*n*) for each experimental group/condition, given as a discrete number and unit of measurement
☒	☐	A statement on whether measurements were taken from distinct samples or whether the same sample was measured repeatedly
☒	☐	The statistical test(s) used AND whether they are one- or two-sided<br>*Only common tests should be described solely by name; describe more complex techniques in the Methods section.*
☒	☐	A description of all covariates tested
☒	☐	A description of any assumptions or corrections, such as tests of normality and adjustment for multiple comparisons
☒	☐	A full description of the statistical parameters including central tendency (e.g. means) or other basic estimates (e.g. regression coefficient) AND variation (e.g. standard deviation) or associated estimates of uncertainty (e.g. confidence intervals)
☒	☐	For null hypothesis testing, the test statistic (e.g. *F*, *t*, *r*) with confidence intervals, effect sizes, degrees of freedom and *P* value noted<br>*Give P values as exact values whenever suitable.*
☒	☐	For Bayesian analysis, information on the choice of priors and Markov chain Monte Carlo settings
☒	☐	For hierarchical and complex designs, identification of the appropriate level for tests and full reporting of outcomes
☒	☐	Estimates of effect sizes (e.g. Cohen's *d*, Pearson's *r*), indicating how they were calculated

Our web collection on statistics for biologists contains articles on many of the points above.

Software and code

Policy information about availability of computer code

Data collection	We performed a descriptive analysis of study characteristics and venous ultrasound assessment characteristics during spaceflight with a special focus on studies reporting on deep vein thrombosis screening.We provided a summary of findings (in-flight changes), the effect of venous interventions on these changes, and the appropriateness of venous ultrasound assessment in spaceflight in comparison with terrestrial standards.<br>We did not report a quantitative synthesis of the changes and the effects of interventions as this was not the primary objective and because of an important heterogeneity between studies.<br>No software was used
Data analysis	No software was used

For manuscripts utilizing custom algorithms or software that are central to the research but not yet described in published literature, software must be made available to editors and reviewers. We strongly encourage code deposition in a community repository (e.g. GitHub). See the Nature Portfolio guidelines for submitting code & software for further information.

Data

Policy information about availability of data

All manuscripts must include a data availability statement. This statement should provide the following information, where applicable:

- Accession codes, unique identifiers, or web links for publicly available datasets
- A description of any restrictions on data availability
- For clinical datasets or third party data, please ensure that the statement adheres to our policy

All additional information available in Supplementary Material. No code access or other access needed.

Research involving human participants, their data, or biological material

Policy information about studies with human participants or human data. See also policy information about sex, gender (identity/presentation), and sexual orientation and race, ethnicity and racism.

Reporting on sex and gender	This information has not been collected on individuals. This is a systematic review of aggregated data. Sex and gender were reported as in the original publications.
Reporting on race, ethnicity, or other socially relevant groupings	This information has not been collected on individuals. This is a systematic review of aggregated data.
Population characteristics	See above
Recruitment	Selection of studies and not individuals.
Ethics oversight	Not relevant to a systematic review

Note that full information on the approval of the study protocol must also be provided in the manuscript.

Field-specific reporting

Please select the one below that is the best fit for your research. If you are not sure, read the appropriate sections before making your selection.

☒ Life sciences ☐ Behavioural & social sciences ☐ Ecological, evolutionary & environmental sciences

For a reference copy of the document with all sections, see nature.com/documents/nr-reporting-summary-flat.pdf

Life sciences study design

All studies must disclose on these points even when the disclosure is negative.

Sample size	Not relevant to the study (a systematic review)
Data exclusions	Selection criteria (1) the study had to have been carried-out in human participants, (2) in the setting of short or long-term spaceflights or acute exposure to microgravity, (3) using an US method including B-mode US, Doppler US, duplex US or Colour-Doppler US modality and (4) reporting any US assessment of the neck/ upper limb or lower limb venous systems (Table 1). Data exclusions: Studies on animals, human studies on ground-based spaceflight analogues, reviews, and duplicates (i.e., same record found in different databases) were excluded.
Replication	Study selection/ assessment was performed by two independent and blinded reviewers to avoid selection bias. Disagreements were all resolved through discussion between the assessors.
Randomization	Not relevant to the study. It is not a clinical trial.
Blinding	Not relevant to the study. It is not a clinical trial.

Reporting for specific materials, systems and methods

We require information from authors about some types of materials, experimental systems and methods used in many studies. Here, indicate whether each material, system or method listed is relevant to your study. If you are not sure if a list item applies to your research, read the appropriate section before selecting a response.

Materials & experimental systems

n/a	Involved in the study
☒	☐ Antibodies
☒	☐ Eukaryotic cell lines
☒	☐ Palaeontology and archaeology
☒	☐ Animals and other organisms
☒	☐ Clinical data
☒	☐ Dual use research of concern
☒	☐ Plants

Methods

n/a	Involved in the study
☒	☐ ChIP-seq
☒	☐ Flow cytometry
☒	☐ MRI-based neuroimaging

Plants

Seed stocks	Not applicable
Novel plant genotypes	Not applicable
Authentication	Not applicable
